# Estimated hospitalisations attributable to seasonal and pandemic influenza in Australia: 2001- 2013

**DOI:** 10.1371/journal.pone.0230705

**Published:** 2020-04-13

**Authors:** Aye M. Moa, David J. Muscatello, Robin M. Turner, C. Raina MacIntyre

**Affiliations:** 1 Biosecurity Program, The Kirby Institute, University of New South Wales, New South Wales, Sydney, Australia; 2 School of Public Health and Community Medicine, Faculty of Medicine, University of New South Wales, New South Wales, Sydney, Australia; 3 Biostatistics Unit, Dean’s Office Dunedin School of Medicine, University of Otago, Dunedin, New Zealand; 4 College of Public Service & Community Solutions, Arizona State University, Phoenix, Arizona, United States of America; Public Health Agency of Canada retiree, CANADA

## Abstract

**Background:**

Influenza continues to cause seasonal epidemics and pandemics in humans. The burden of influenza is underestimated by traditional laboratory-based surveillance, and modelled estimates are required for influenza-attributable morbidity and mortality. We aimed to estimate the influenza-attributable hospitalisation in Australia, by influenza type.

**Methods:**

A generalised-additive regression model was used to estimate type- and age-specific influenza-attributable hospitalisation rates per 100,000 population by principal diagnosis in Australia, from 2001 through 2013. Weekly counts of laboratory-confirmed influenza notifications and by type, influenza A and B were used as covariates in the model. Main principal diagnosis categories of interest were influenza and pneumonia and respiratory admissions. A smoothing spline was used to control for unmeasured time varying factors. Results for 2009, in which the pandemic influenza A(H1N1)pdm09 virus circulated, were not included in annual averages and are reported separately.

**Results:**

During the study period, the estimated annual average, all-age, annual respiratory hospitalisation rates attributable to seasonal influenza type A, B and total influenza were 45.4 (95% CI: 34.9, 55.9), 32.6 (95% CI: 22.8, 42.4), and 76.9 (95% CI: 73.6, 80.2) per 100,000 population, respectively. During 2009, the estimated total pandemic influenza-attributable, all-age, respiratory hospitalisation rate was 56.1 (95% CI: 47.4, 64.9) per 100,000. Older adults (≥85 years of age) experienced the highest influenza-attributable hospitalisation rates for both seasonal and 2009 pandemic influenza. Collinearity between influenza A and B time series in some years limited the ability of the model to resolve differences in influenza attribution between the two virus types.

**Conclusion:**

Both seasonal and pandemic influenza caused considerable morbidity in Australia during the years studied, particularly among older adults. The pandemic hospitalisation rate in 2009 was lower than the average overall annual rate for seasonal influenza, but young to middle aged adults experience a hospitalisation rate similar to that of severe seasonal influenza.

## Introduction

Persons of all ages are susceptible to influenza infection [1, 2]. Influenza is a vaccine preventable disease, and yet the burden of influenza remains high, varying from season to season, with pandemics being an unpredictable but constant risk [3–5].

Influenza infection is a common illnesses and annual incidence of infection is not captured accurately by routine surveillance methods such as laboratory-confirmed infections, and thus true estimates of morbidity and mortality attributable to influenza is not known [6]. Statistical modelling has therefore been used to estimate the true burden of disease impact attributable to influenza infection [3, 4, 7, 8]. Although influenza typically causes acute respiratory illness, it can also contribute to, or exacerbate, other severe acute or chronic illnesses. Therefore, statistical approaches to estimating morbidity and mortality attributable to influenza infection, aim to determine the portion of broader categories of outcomes or diagnoses than just influenza. Typically, a respiratory diagnosis such as influenza combined with pneumonia, all respiratory, and non-respiratory such as all cardiovascular and all-cause diagnosis groups are considered to estimate influenza-attributable illnesses for burden of disease studies [1, 3, 9]. A recent global modelling study estimated that approximately 290,000–650,000 respiratory deaths were attributable to seasonal influenza annually [10].

The only previous all-age study of hospitalisation burden of influenza in Australia is by Newall and Scuffham for the period 1998 through 2005, with an estimated annual average rate of around 94 respiratory hospitalisations per 100,000 population attributable to seasonal influenza infection among persons of all ages [8].

Factors affecting influenza’s impact include virulence of the circulating virus strains, vaccine coverage and effectiveness, and seasonal vaccine mismatch [11]. Australia has a funded a national influenza vaccination program. In Australia since 1999, free influenza vaccination has been available under the national immunisation program for persons aged ≥65 years, and for certain risk groups [12]. The classification of risk groups has varied over time [12, 13]. Influenza vaccination is currently recommended for all persons aged ≥6 months [14]. In a 2014 national survey, it was reported that 73% of older Australians aged ≥65 years received influenza vaccination under the national scheme [15]. An estimated seasonal influenza vaccination coverage of 23% was reported in adults aged 18–64 years in 2009, and a reduced influenza vaccine uptake with an estimated vaccination rate of 36% was observed among high-risk individuals, mostly in adults 18–64 years of age [16].

The majority of Australia’s population lives in a temperate zone, and overall influenza occurrence is moderately seasonal [17]. Due to the changing epidemiology of influenza and varied influenza vaccine effectiveness from season to season, estimates of burden of influenza infection need to be updated frequently to inform effective influenza prevention and vaccination programs as well as to provide data for cost effectiveness studies. Our study aims to estimate total hospitalisations attributable to seasonal influenza and for the pandemic year, 2009 by virus type and age in Australia. This study would provide recent estimates of influenza-attributable hospitalisations in Australia for the period July 2001 –December 2013.

## Materials and methods

### Study design and setting

This was a retrospective study using a complete national hospital morbidity database in Australia, between July 2001 and June 2014. We report influenza-attributable hospitalisations for 13 years from 2001 through 2013, and results for the pandemic year 2009 are reported separately.

### Data sources

#### Hospital admissions data

Weekly counts of national hospitalisations data, aggregated to weeks by date of admission, were obtained retrospectively from the National Hospital Morbidity Database (NHMD), the Australian Institute of Health and Welfare (AIHW), from the first week of July 2001 to the last week of June 2014. Hospitalisation diagnoses in Australia during the study period were coded using the ICD-10-AM edition for individual year [18]. The clinical diagnosis coding used for ICD-10-AM is reviewed and modified annually in Australia. The NHMD contained almost all hospital records in Australia including both public and private hospital separations for the study period. The counts were aggregated under the following principal diagnosis categories from the ICD-10-AM: influenza and pneumonia (J09−J18) and all respiratory disease (J00–J99). For each category, data were aggregated for persons of all ages, and for the following age groups: 0–14, 15–64, 65–84, and ≥85 years.

#### National notifiable diseases surveillance data

To provide a proxy indicator of relative week to week changes in influenza notifications in the model, we obtained national weekly counts of laboratory-confirmed influenza notifications from the National Notifiable Diseases Surveillance System, the Department of Health (NNDSS) [19].

#### Population data

To calculate population rates, national resident population estimates from the Australian Bureau of Statistics (ABS) was used [20]. Weekly population estimates were obtained by linear interpolation of the annual estimates.

### Statistical methods

Semi-parametric generalised-additive models (GAM) with a smoother of time were used to model weekly age-specific hospitalisation rate per 100,000 population (outcome) with weekly counts of all-age influenza notifications as an independent variable. The choice of GAM over ordinary parametric regression with harmonic (sinusoidal) smoothers was chosen because harmonic models with sinusoidal seasonality require an assumption that background variability in the hospital time series is perfectly seasonal. The harmonic approach requires an assumption that all diseases other than influenza that contribute to the hospital time series are perfectly seasonal. Using a more flexible smoother of time relaxes this assumption. This may also lead to better control of autocorrelation in model residuals meaning that the modelling assumption of independent residuals is better met. GAM has been applied in several studies of infectious disease outcomes, including influenza [21–24].

To estimate the separate effect of influenza types A and B, weekly notification counts of each type were entered into the model, with a separate independent variable for each influenza type included for each year. For a given year, these variables included notification counts for that year, but counts were set to zero in all other years. This allowed us to control for substantial variation over time in influenza testing practices by healthcare providers, as well as variation from year to year in virulence of viral strains, vaccination coverage or vaccine effectiveness.

We examined the data for holiday periods using box whisker plots of the distribution. Holidays included were Christmas Day, New Year’s Day, Australia Day, Easter, and ANZAC day. Other holiday periods were a week before Christmas, a week before Easter, and two and three weeks after the New Year, which are within the main summer school holiday period in Australia. A separate indicator time series variable was created for each holiday valued at 0 for a non-holiday week and 1 for a holiday week.

The model equation for influenza type-specific estimates was:

$$\begin{array}{ll} Y= & \beta_{0}+\sum_{year=2001}^{2014}\beta_{1,year}\left(\text{Influenza}\;{\text{A}}_{\text{year}}\right)+\sum_{year=2001}^{2014}\beta_{2,year}\left(\text{Influenza}\;{\text{B}}_{\text{year}}\right)\\ & \begin{array}{l} +\beta_{3}\left(\text{week}\;\text{before}\;\text{Xmas}\right)+\beta_{4}\left(\text{Xmas}\;\text{week}\right)+\beta_{5}\left(\text{new}\;\text{year}’\text{s}\;\text{week}\right)\\ +\beta_{6}\left(\text{one}\;\text{week}\;\text{after}\;\text{new}\;\text{year}\right)+\beta_{7}\left(\text{two}\;\text{weeks}\;\text{after}\;\text{new}\;\text{year}\right)\\ +\beta_{8}\left(\text{three}\;\text{weeks}\;\text{after}\;\text{new}\;\text{year}\right)+\beta_{9}\left(\text{week,}\;\text{Australia}\;\text{day}\right)\\ +\beta_{10}\left(\text{one}\;\text{week}\;\text{before}\;\text{Easter}\right)+\beta_{11}\left(\text{Easter}\;\text{week}\right)+\beta_{12}\left(\text{week,}\;\text{ANZAC}\;\text{Day}\right)\\ +\beta_{13}\left(\text{time}\right)+\text{spline}\left(\text{time}\right)+\text{error}\left(\text{time}\right) \end{array} \end{array}.$$

where *Y* represents the weekly hospitalisation rate per 100,000 population for a specific age group and disease category. β_0_ is the model intercept and *β*_1,*year*_, *β*_2,*year*_, *β*_3_, *β*_4_, …, *β*_12_ represent the values of parameter estimates of the respective independent or indicator variable. *β*_13_ (time) is a linear term for week number to control for any long-term (ecological) linear time trend in the hospitalisation rate, and spline (time) is a smoothing spline of the week number to control for unmeasured time varying factors such as non-influenza-related seasonal variation in the hospitalisation categories.

For the smoothing spline of week number, we used a total of 79 degrees of freedom (DF), which includes 6 turning points in a year for the flexibility of the smoothing spline plus an additional 1 DF for linear term in the model. This a priori choice of degrees of freedom was based on providing a smoother of time that provided a balanced approach. It is longer than the time scale of weekly influenza-attributable variation that we are interested in, while conservatively limiting autocorrelation in the model residuals. Limiting autocorrelation improves the ability of the model to meet the assumption of independent residuals.

For estimating the total influenza hospitalisation rate, the same model equation was used except with total weekly influenza notifications rather than notifications by type.

The estimated influenza-associated weekly hospitalisation rate in each disease category and age group was calculated by multiplying the corresponding parameter estimates by the number of influenza notifications (both A and B and total) in the week. Weekly influenza-attributable count estimates were obtained by multiplying weekly rate estimates by the population estimate in that week. Annual total rates and counts were calculated from the weekly estimates.

Results for the pandemic year 2009 due to the influenza A(H1N1)pdm09 virus are reported separately from seasonal influenza here. Since influenza B comprised less than one per cent of influenza notifications in 2009 in Australia [25], only results for the total influenza model are reported in that year and treated as results for the pandemic virus.

The 95% confidence interval (CI) was obtained using the equation as described below.

β±(1.96xSE),

where SE is the standard error of the parameter estimate from the model.

Simple averages of annual seasonal influenza hospitalisation rates were calculated, excluding the 2009 pandemic year. The standard errors for calculating the CI for average hospitalisation rates across studied years were calculated by using the square root of the sum of the squared standard errors of parameter estimates for each annual influenza variable, divided by the number of influenza seasons included in the average, as shown using the formula below.

$$\text{SE}=\sqrt{SE_{1}^{2}+..+SE_{n}^{2}}/12,$$

where *SE*_1_… *SE*_*n*_ are the standard errors of the parameter estimates for the influenza variables in each of the n years averaged.

### Model fit

The normality of model residuals was checked using quantile-quantile (QQ) plots of the residuals. Autocorrelation of time series data in the model residuals were also checked using autocorrelation plots of the model residuals.

### Sensitivity analyses

We conducted two sensitivity analyses. First, we tested the effect of altering the flexibility of the spline, running the GAM with each of 4 (less flexible) and 8 knots (more flexible) per year for the all-age respiratory hospitalisation category by influenza type. Second, we examined the difference in estimates that would be obtained using a more conventional harmonic ordinary linear regression model with annual sinusoidal seasonal time smoothing for the all-age respiratory hospitalisation category by influenza type.

All analyses were performed using the SAS Enterprise Guide (SASEG) version (6.1) [26]. Ethics approval for the study was obtained from the University of New South Wales, reference number (HREA–Ref: 2014-7-62). The hospital and influenza notification data was provided as aggregate weekly counts, which prevented identification of individual persons.

## Results

### Characteristics of study data

From July 2001 through December 2013, 4.5 million respiratory disease and 0.9 million influenza and pneumonia admissions in persons of all ages were recorded during the study period.

By age group, observed weekly mean hospitalisation rates were highest in persons aged ≥85 years and over with a rate of 64.9 and 162.2 per 100,000 population in influenza and pneumonia, and respiratory diagnosis groups, respectively. This was followed by the 65–84 years of age, with a weekly mean rate of 19.8 and 75.5 per 100,000, respectively. In children aged <15 years, the highest cause-specific weekly mean hospitalisation rate was seen in respiratory diagnoses, with a rate of 47.3/100,000 (S1 Table).

**Fig 1:**
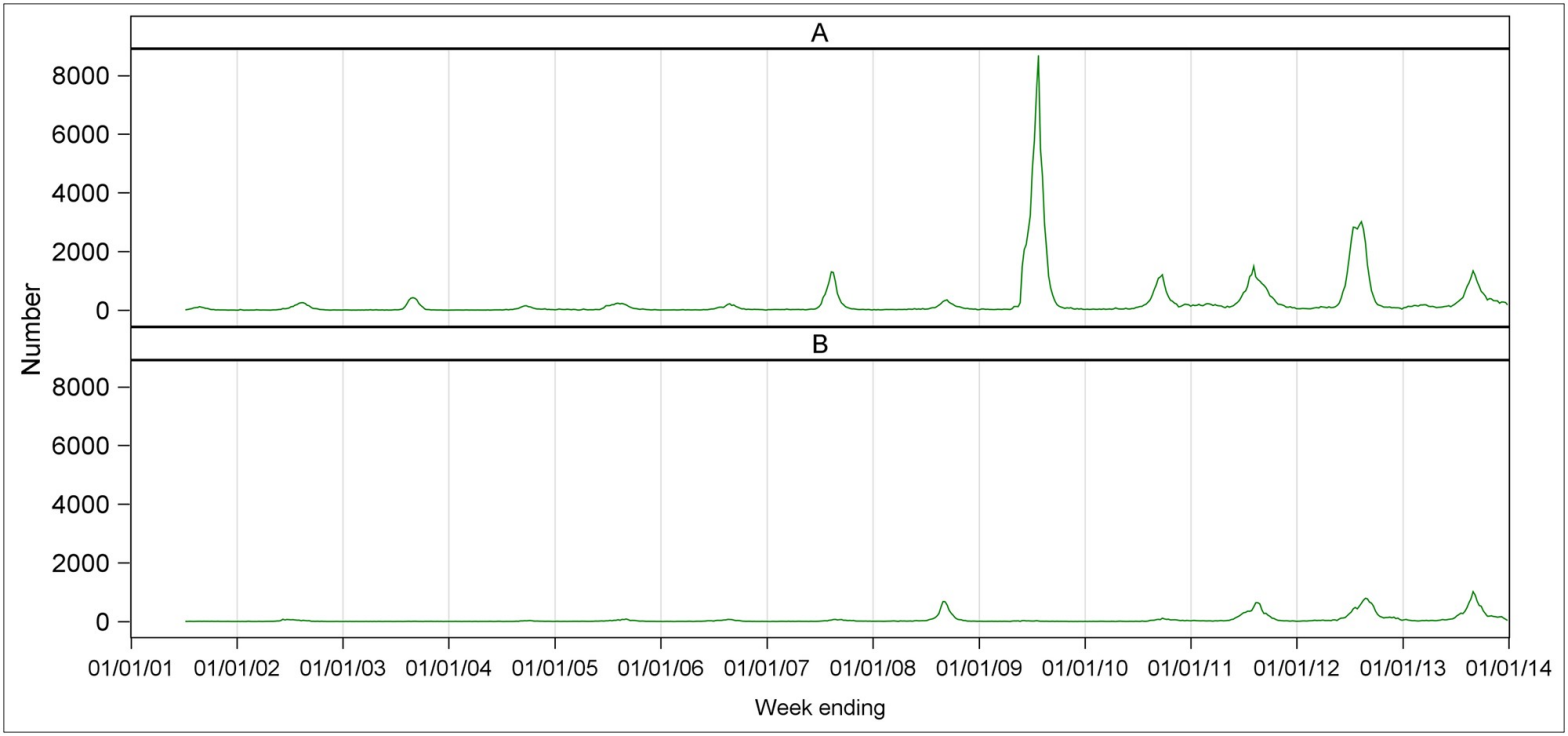
Weekly number of laboratory-confirmed notifications of influenza A and influenza B by year, Australia, 2001–2013.

Among the 208,630 total influenza notifications during the study period, influenza A contributed the highest number. A total of 169,259 (81.1%) influenza A and 39,371 (18.9%) influenza B notifications were received nationally (S2 Table). The overall weekly mean number of notifications was 260 for influenza A and 60 for influenza B. There was great variation from year to year, and increased notifications were seen especially in the post-pandemic (post-2009) years. The highest number of influenza A notifications was during the pandemic year 2009, while influenza B reached its highest counts in 2012 and 2013. For influenza A, the highest annual mean count was 1123 in 2009 and the lowest counts was 30 in 2004. For influenza B, the highest annual mean count was 203 in 2012 and the lowest was 2 in 2004 (S2 Table). The corresponding time series of laboratory-confirmed influenza A and B notifications are presented in Fig 1.

**Fig 2:**
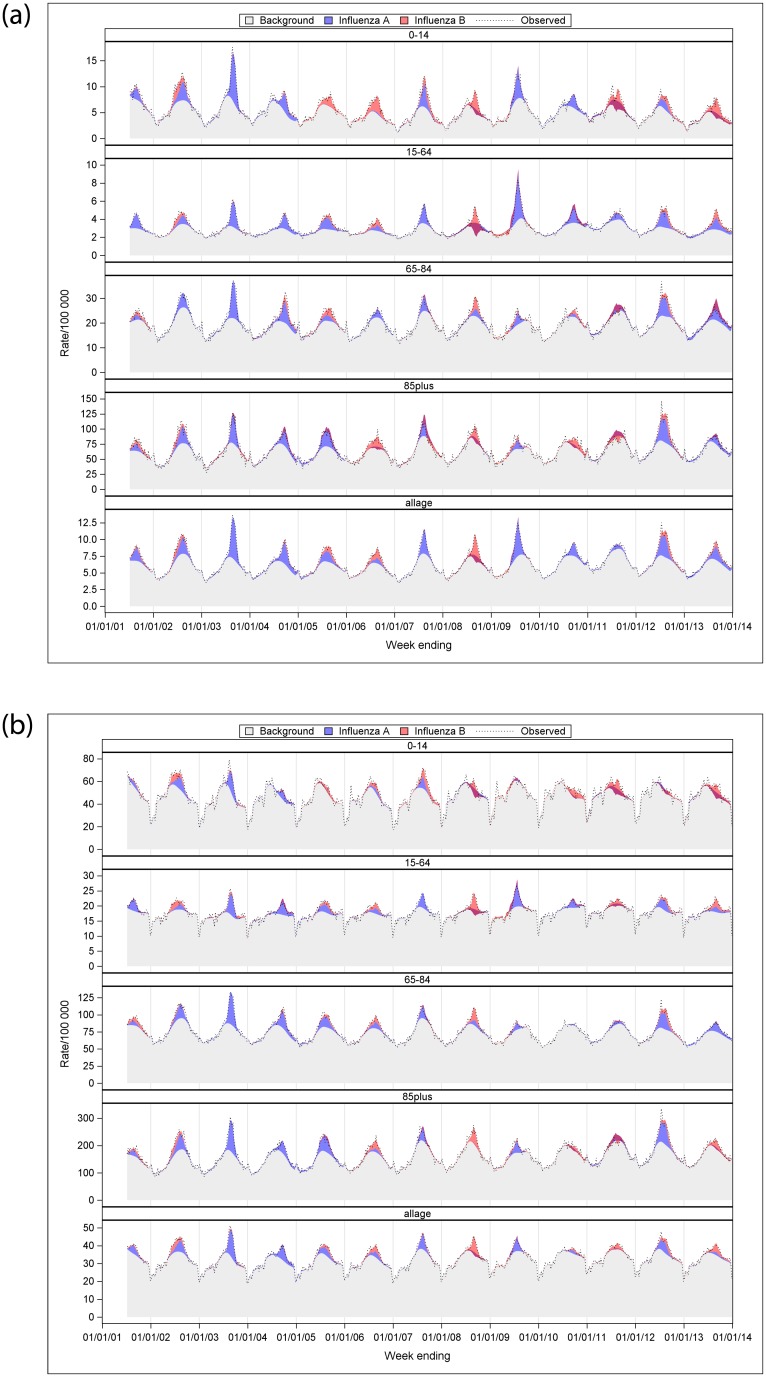
a. Estimated, observed and baseline influenza-attributable influenza and pneumonia hospitalisation rate per 100,000 population by age group and year, Australia, 2001–2013. b. Estimated, observed and baseline influenza-attributable respiratory hospitalisation rate per 100,000 population by age group and year, Australia, 2001–2013.

**Fig 3:**
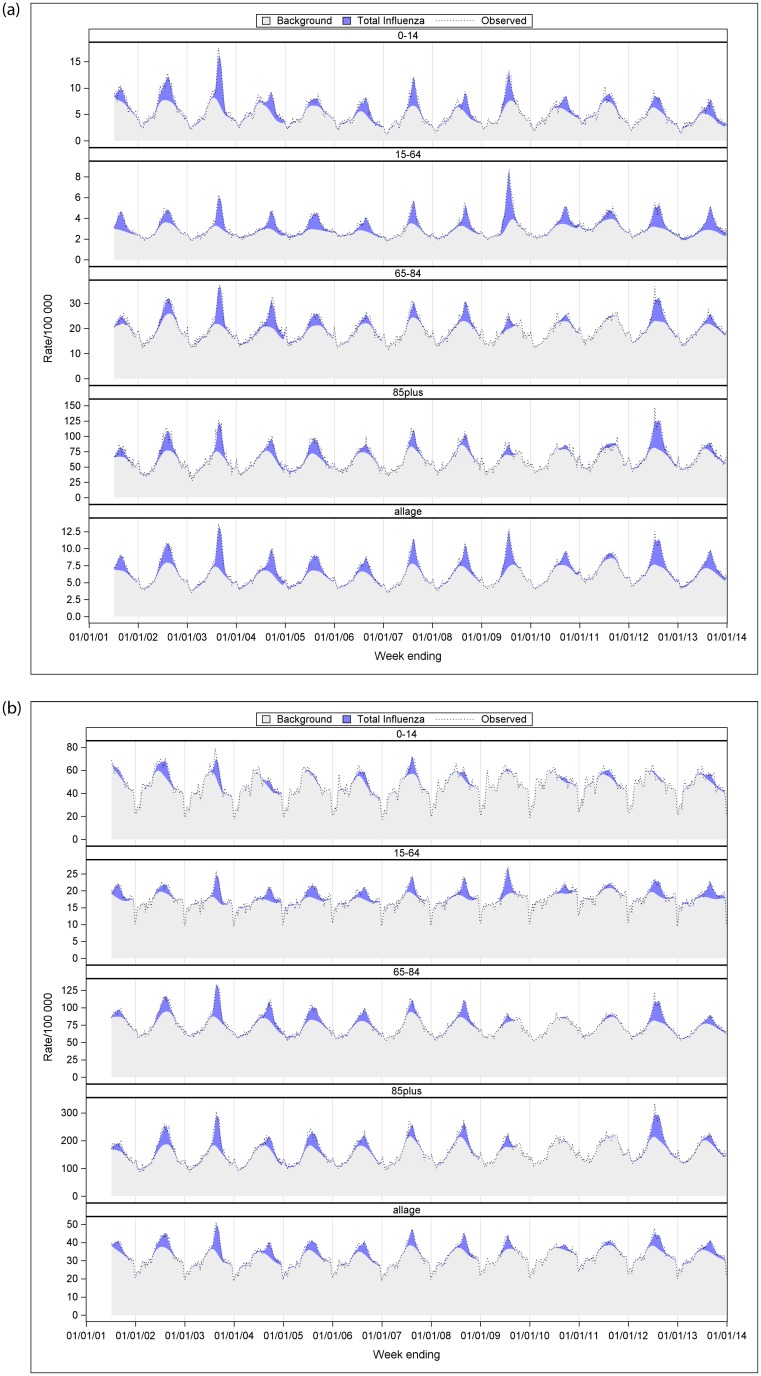
a. The total influenza model—Estimated, observed and baseline influenza-attributable influenza and pneumonia hospitalisation rate per 100,000 population by age group and year, Australia, 2001–2013. b. The total influenza model—Estimated, observed and baseline influenza-attributable respiratory hospitalisation rate per 100,000 population by age group and year, Australia, 2001–2013.

Fig 2a and 2b showed a time series of type- and age-specific influenza-attributable influenza and pneumonia and respiratory hospitalisation rate per 100,000 population. Over the 13 influenza seasons, influenza A-attributable both influenza and pneumonia and respiratory hospitalisations were most distinct in 2003, 2009 and 2012. Similarly, relatively higher influenza B-attributable respiratory hospitalisations were seen in 2008 and 2013. In Fig 2a and 2b, darker red areas reflect negative estimates of influenza A or B from the model. There was collinearity between influenza A and B time series in some years that limited the ability of the model to resolve differences in influenza attribution between the two virus types. For influenza A, this occurred in 2008, 2011 to 2013, and was more pronounced in <15 year-olds. For influenza B, this was pronounced in 2011. Time series graphs of age-specific total influenza-attributable hospitalisation rates for influenza and pneumonia, and respiratory are described in Fig 3a and 3b.

### Seasonal influenza-attributable hospitalisations by year

Annual estimates of influenza A, B and total influenza-attributable hospitalisations by principal diagnosis and age group are provided in the (S3 and S4 Tables). The highest annual estimated rate per 100,000 in persons of all ages influenza A-attributable respiratory hospitalisation was 111.8 (95% CI: 101.0, 122.7) in 2003, followed by 92.2 (95% CI: 58.4, 125.9) in 2004, 66.8 (95% CI: 51.2, 82.3) and 66.8 (95% CI: 47.7, 85.8) in 2002 and 2012 respectively. The highest influenza B-attributable respiratory hospitalisation rate was 77.3 (95% CI: 49.5, 105.1) per 100,000 population in 2008 (S3 Table). For total influenza, the highest all-age influenza-attributable respiratory hospitalisation rate per 100,000 persons was 120.7 (95% CI: 112.1, 129.4) in 2003, followed by 98.0 (95% CI: 86.3, 109.7) and 97.2 (95% CI: 85.9, 108.5) in 2002 and 2012 respectively (S3 Table).

### Mean annual seasonal influenza-attributable hospitalisations

Table 1 presents the estimated annual average hospitalisation rates per 100,000 population and counts by virus type, principal diagnosis and age group over the study period, excluding the 2009 pandemic year. For all-age respiratory hospitalisations, the annual average total influenza-attributable population rate was 76.9 (95% CI: 73.6, 80.2) per 100,000. The rates for influenza A and B were 45.4 (95% CI: 34.9, 55.9) and 32.6 (95% CI: 22.8, 42.4) per 100,000, respectively. Influenza B accounted for 42% of the estimated all-age total influenza rate. By age group, the highest estimated rate of total influenza-attributable hospitalisation per 100,000 was 510.3 (95% CI: 484.8, 535.8) in persons aged ≥85 years. The rate per 100,000 declined with age to 43.5 (95% CI: 41.4, 45.5) in 15–64 year-olds, and increased again to 76.9 (95% CI: 66.8, 87.1) in <15 year-olds. Graphs for all-age, estimated average annual rate of influenza-attributable hospitalisations from influenza and pneumonia and respiratory diagnosis categories, using influenza A and B model and the total model are also presented in the (S1 and S2 Figs).

**Table 1:** Estimated average annual seasonal influenza-attributable hospitalisation rate per 100,000 population and count, by influenza type, principal diagnosis and age group, Australia, 2001–2013^a^.

Principal diagnosis Influenza type	Age group									
	0–14 years		15–64 years		65–84 years		≥85 years		All-age	
	Rate (95% CI)	Count (95% CI)	Rate (95% CI)	Count (95% CI)	Rate (95% CI)	Count (95% CI)	Rate (95% CI)	Count (95% CI)	Rate (95% CI)	Count (95% CI)
Influenza and pneumonia										
Influenza A	**15.5 (10.6, 20.4)**	**615 (410, 820)**	**13.0 (11.4, 14.6)**	**1,834 (1,596, 2,071)**	**58.5 (48.3, 68.6)**	**1,459 (1,188, 1,730)**	**203.7 (159.6, 247.8)**	**680 (508, 852)**	**21.9 (19.4, 24.4)**	**4,575 (4,027, 5,122)**
Influenza B	**21.5 (17.0, 26.1)**	**892 (702, 1,082)**	**5.1 (3.6, 6.7)**	**731 (512, 950)**	8.3 (-1.2, 17.8)	177 (-71, 425)	23.6 (-17.7, 64.9)	82 (-73, 238)	**9.0 (6.6, 11.3)**	**1,892 (1,386, 2,397)**
Total influenza	**37.1 (35.5, 38.7)**	**1,513 (1,449, 1,577)**	**19.5 (19.0, 20.0)**	**2,762 (2,688, 2,836)**	**64.2 (61.0, 67.4)**	**1,561 (1,480, 1,641)**	**236.6 (222.4, 250.8)**	**784 (733, 834)**	**31.6 (30.8, 32.4)**	**6,619 (6,452, 6,786)**
Respiratory										
Influenza A	12.5 (-19.6, 44.6)	430 (-922, 1,781)	**25.9 (19.4, 32.3)**	**3,575 (2,627, 4,523)**	**168.2 (143.7, 192.7)**	**4,108 (3,456 4,760)**	**380.1 (299.6, 460.6)**	**1,224 (911, 1,537)**	**45.4 (34.9, 55.9)**	**9,281 (6,977, 11,584)**
Influenza B	**76.4 (46.4, 106.3)**	**3,170 (1,917, 4,424)**	**15.7 (9.7, 21.7)**	**2,258 (1,382, 3,133)**	**45.1 (22.2, 68.0)**	**1080 (482, 1,677)**	**115.0 (39.7, 190.2)**	**409 (125, 692)**	**32.6 (22.8, 42.4)**	**6,952 (4,826, 9,079)**
Total influenza	**76.9 (66.8, 87.1)**	**3,122 (2,703, 3,540)**	**43.5 (41.4, 45.5)**	**6,123 (5,832, 6,414)**	**210.5 (202.8, 218.2)**	**5108 (4,915, 5,301)**	**510.3 (484.8, 535.8)**	**1,690 (1,600, 1,780)**	**76.9 (73.6, 80.2)**	**16,038 (15,337, 16,739)**

^a^ The pandemic year 2009 was excluded from averages, and both statistically significant and non-significant estimates were included in the average.

Statistically significant estimates are shown in bold.

CI = confidence interval.

The estimated all-age total influenza-attributable pneumonia and influenza hospitalisation rate was 31.6 (95% CI: 30.8, 32.4) per 100,000, representing 41% of the estimated respiratory hospitalisation rate. Like respiratory hospitalisations, the estimated pneumonia and influenza hospitalisation rate per 100,000 declined with age, from 236.6 (95% CI: 222.4, 250.8) to 19.5 (95% CI: 19.0, 20.0) in 15–64 year-olds, and then increased somewhat to 37.1 (95% CI: 35.5, 38.7). Influenza B accounted for 28% of the all-age total influenza rate in persons of all ages.

### Pandemic influenza-attributable hospitalisations, 2009

For the influenza A(H1N1)pdm09 pandemic year of 2009, hospitalisation rates of 56.1 (95% CI: 47.4, 64.9) and 38.9 (95% CI 36.8, 41.0) per 100,000 were estimated for respiratory and influenza and pneumonia principal diagnosis groups, respectively. Pneumonia and influenza principal diagnoses accounted for 69% of the all-age respiratory hospitalisation rate. Hospitalisation rates were highest in persons aged ≥85 years, with 323.9 (95% CI: 256.2, 391.6) per 100,000 for respiratory and 125.5 (95% CI: 87.8, 163.2) per 100,000 population for influenza and pneumonia. The respiratory estimate for 0–14 year-olds was not statistically significant, but the influenza and pneumonia estimate was significant, at 41.4 (95% CI: 37.3, 45.5) per 100,000 (Table 2). The pandemic rate in 15–64 year-olds was similar to the estimate for seasonal influenza in some year in that age group (S3 Table).

**Table 2:** Estimated pandemic influenza A(H1N1)pdm09 virus hospitalisation rate per 100,000 population and count, by principal diagnosis and age group, Australia, 2009.

	Age group									
	0–14 years		15–64 years		65–84 years		≥85 years		All-age	
Principal diagnosis	Rate (95%CI)	Count (95%CI)	Rate (95%CI)	Count (95%CI)	Rate (95%CI)	Count (95%CI)	Rate (95%CI)	Count (95%CI)	Rate (95%CI)	Count (95%CI)
Influenza and pneumonia	**41.4 (37.3, 45.5)**	**1,722 (1,550, 1,894)**	**36.2 (34.8, 37.6)**	**5,306 (5,104, 5,508)**	**37.5 (29.0, 46.1)**	**948 (733, 1,164)**	**125.5 (87.8, 163.2)**	**463 (324, 602)**	**38.9 (36.8, 41.0)**	**8,438 (7,984, 8,892)**
Respiratory	14.1 (-12.9, 41.1)	586 (-536, 1708)	**57.2 (51.7, 62.6)**	**8,374 (7,578, 9,170)**	**80.5 (60.0, 101.0)**	**2,034 (1,517, 2,551)**	**323.9 (256.2, 391.6)**	**1,196 (946, 1,445)**	**56.1 (47.4, 64.9)**	**12,185 (10,280, 14,091)**

Since influenza B comprised <1% of influenza notifications in 2009, results from the total influenza model are reported here for the influenza A (H1N1)pdm09 virus.

Statistically significant estimates are shown in bold.

CI = confidence interval.

### Model fit

The Q-Q plots showed some minor departures from normality in the model residuals, particularly at the extremities of the line of normality. Autocorrelation plots of the residuals indicated low to moderate autocorrelation, varying by diagnosis category. Furthermore, we examined correlation between the influenza A and B time series in each year. Influenza A and B were highly correlated, with correlation coefficients ≥ 0.9, in 2004, 2006, 2008, 2011 and 2013.

### Sensitivity analyses

Varying the number of knots to 4 or 8 per year in the smoothing spline or using a harmonic ordinary linear regression model produced estimates for average annual, all-age influenza A- and B-attributable respiratory hospitalisation rates with overlapping confidence intervals (S5 Table). Autocorrelation plots produced from the GAM models for all-age respiratory hospitalisations using 4, 6 and 8 spline knots per year and for the harmonic model are provided in the (S3 Fig). The autocorrelation plots for 4 knots/year smoothing showed weak but statistically significant positive, first order autocorrelation with 4 knots/year, which became weaker but still significant for 6 knots/year and minimal and non-significant with 8 knots per year. However, with 8 knots/year, some weak, significant higher order negative correlations were observed. With the harmonic model, moderate first order and weak second order autocorrelation were apparent (S3 Fig).

## Discussion

We estimated that from 2001 through 2013, seasonal influenza was associated with an average annual, all-age respiratory hospitalisation rate of 77 per 100,000 population, of which influenza B accounted for 42% of the rate. For influenza and pneumonia hospitalisations, the rate was about 32 per 100,000 and influenza B accounted for 28% of that rate. Rates for both disease categories were lowest in 15–64 year-olds, and increased with age, with the highest rates in ≥85 year-olds. Rates in children for both principal diagnosis categories were almost double that of 15–64 year-olds. Collinearity between influenza A and B surveillance time series in several years limited the models’ ability to resolve influenza type-specific contributions to the hospital burden particularly in children.

For the 2009 pandemic year, we estimated an all-age influenza A(H1N1)pdm09-attributable respiratory hospitalisation rate of 56 per 100,000, which was lower than the average annual total seasonal influenza-attributable rate. On the other hand, the influenza and pneumonia pandemic rate of around 39 per 100,000 was higher than the annual average seasonal influenza rate for influenza and pneumonia. These differences are explained by higher rates in the 15–64 years age group and lower rates in other adult age groups for pandemic compared with seasonal influenza. Even so, the rate in 15–64 year-olds was not outstanding when compared with seasonal influenza in some specific years. Among children aged under 15 years, only the influenza and pneumonia hospitalisation rate was statistically significant (about 41 per 100,000) for pandemic influenza and this was similar to the estimated average annual total seasonal influenza-attributable influenza and pneumonia hospitalisation rate in that age group.

During the 2003 and 2012 influenza seasons, influenza A(H3N2) dominated and influenza A notifications increased. These were recognised as severe influenza seasons in Australia [27–30]. During these years, our study showed increased respiratory hospitalisation rates in adults aged 65 years and over attributable to influenza A or total influenza. Our study showed that the 2003 influenza season was the worst season for hospitalisations among the years studied. Consistent with other studies [8, 31–33], hospitalisation rates per 100,000 population for seasonal influenza were high among older adults in our study, with the highest rates in persons aged ≥85 years. This is in contrast to laboratory-confirmed notifications data from the Department of Health, which show higher frequency in children compared with other age groups [34], possibly reflecting a testing bias.

The finding of relatively high hospitalisation rates in 15–64 year-olds for pandemic compared with seasonal influenza is consistent with the age shift typically seen with pandemic influenza and seen in 2009 [5]. Despite popular views that the 2009 pandemic was not severe in terms of mortality [35], the pandemic year showed an increased hospitalisation rate in 15–64 year-olds in our study. The rate of respiratory hospitalisations in persons of all ages in 2009 (56 per 100,000 population) in our study was identical to the finding of a Canadian study by Schanzer et al. [7].

There is only one prior study on the all-age burden of influenza-related hospitalisations for Australia. The study by Newall and Scuffham reported a similar age distribution of hospitalisation rates for both influenza and pneumonia, and respiratory illnesses [8]. For all-age hospitalisations, that study reported a population rate of 94 and 42 per 100,000/year for respiratory, and influenza and pneumonia, respectively, somewhat higher than our findings. A New Zealand study found estimates somewhat lower than ours for the period 1994 through 2008 [32]. Differences in study findings may be due to differing modeling methods, influenza vaccine coverage or effectiveness, or epidemic severity in the respective study periods.

The previous Australian study included only total influenza-attributable hospitalisations, thus influenza type-specific comparisons could not be made [8]. There are two recently published studies reporting type-specific hospitalisation burden of influenza, from the United Kingdom (UK) and the United States (US) [31, 36]. While the results of the US study cannot be compared directly due to differences in study timing, diagnosis category and statistical methods used, results were broadly similar for all-age hospitalisations attributable to total influenza: 64 per 100,000 population for respiratory and 35 per 100,000 population for influenza and pneumonia. The US study reported a lower all-age rate of influenza B-attributable respiratory hospitalisation (16 versus our 33 per 100,000 population) and a similar rate for influenza and pneumonia (7 versus our 9 per 100,000 population) [36]. The UK study reported an average annual, all-age seasonal influenza A-attributable respiratory hospitalisation rate of around 44 per 100,000, almost identical to our finding. Their influenza and pneumonia finding of 12 per 100,000 was around half our estimate [31].

Many epidemiological studies reported that the incidence of influenza B is higher in children than older adults [25, 37, 38]. Since those studies relied on the results of influenza testing, they may be subject to testing bias, and may not reflect the increasing risk of hospitalisation with age. On average influenza B accounted for around 17% of seasonal influenza notifications in Australia [25]. Our study estimated that influenza B, on average, accounted for about 42% of all-age seasonal influenza-attributable respiratory hospitalisations. The use of quadrivalent vaccines that include two influenza B virus lineages may be beneficial in reducing the burden of influenza B in Australia [39].

There are several limitations in the study. Many studies include influenza as a single variable across all years, but in this study we included a separate influenza variable for each year, to avoid the problem of increased testing over time. This markedly reduces the statistical power to detect an association between the influenza and hospitalisation time series. A further challenge of this approach is that the influenza A and B time series in a year may be strongly correlated (collinear) thus reducing the ability of the model to resolve each type’s relative contribution to hospitalisations. Therefore, some type-specific results may not be accurate. There are limitations of notifications data in Australia. NNDSS received only laboratory-confirmed cases reported across the country, but laboratories do not report on number of patients tested so proportion positive cannot be obtained. This would avoid the problem of increased testing over time if a proportion was available. Testing and reporting of influenza infection is conducted at the discretion of the medical practitioners or according to local health facility policy. Evidence suggests an increase in testing or reporting after the 2009 pandemic [25] and notification counts have increased year by year. The reason for increased testing is unknown, but may be due to increased awareness of infection by GPs and the public as well as availability of improved diagnostic tools in recent years. Variation in testing practices across the studied years might have influenced our results through varying population biases. However, use of annual variables for influenza may have limited this effect. Another limitation in the study was presence of some negative estimates from the influenza A and B model, which are difficult to interpret. Nevertheless, we included all estimates in the calculation of averages to avoid positive bias in our results. In the majority of years studied, influenza A predominated, which may have led to poor model fit for influenza B. From the sensitivity analysis comparing the use of a proportional allocation of total influenza based on the proportion of influenza detections in year, the difficulty in resolving influenza B’s contribution to hospitalisations was more of a problem in 2008. Influenza B predominated in 2008, but the type-specific model attributed all influenza-attributable hospitalisations to type B. While the proportional approach may be attractive, it may lead to inaccurate conclusions because it does not take account of varying virulence of the virus circulating in a year, or varying susceptibility of the population.

This study only investigated the short-term associations between influenza infection and hospitalisations occurring in the same week, with the burden of longer-term sequelae unable to be estimated. The choice of knots for the smoother of time in our GAM model may have influenced our results. We took a conservative, a priori approach for the choice of knots, which led to some residual autocorrelation, but avoided overfitting the smoother to the observed data. Nevertheless, the sensitivity analysis comparing results between our modelling approach and a more conventional harmonic regression model showed that the results were similar. Further, our choice of knots provided a reasonable level of independence in the model residuals compared with a greater and a lower degree of smoothing and with the harmonic model. Checking of this modelling assumption is often absent from influenza attribution studies. A disadvantage of the harmonic regression model [40] may be that it requires an assumption that all non-influenza diseases that contribute to the diagnosis category being studies have perfect seasonality when aggregated. This is unlikely given that influenza circulated at varying times and with varying epidemic sizes each year. Another limitation is that we were unable to control for other respiratory pathogens, such as respiratory syncytial virus (RSV). RSV laboratory results are not systematically monitored in Australia. This may have led to some misattribution of RSV hospitalisations to influenza [36]. However, a southern hemisphere study from South Africa on influenza and RSV-attributable mortality found that inclusion or exclusion of RSV did not substantially alter the influenza results [41]. Our model fit may have been improved if we had access to hospitalisation data that excluded planned admissions, thus leading to a hospitalisation time series that better reflected acute illness in the population. We were unable to examine influenza A subtypes or influenza B lineages, because subtype is only available for a small proportion of influenza notifications. Due to sparse notification data in some years, we were unable to include age-specific notifications in the age-specific models. Nevertheless, all-age epidemic patterns should reasonably reflect age-specific time series.

To conclude, our study provided more recent estimates of influenza-attributable hospital morbidity in Australia for 12 influenza seasons and a pandemic year. On average, older Australians experienced the highest rate of hospital admissions attributable to both seasonal and pandemic influenza. The majority of influenza-attributable hospitalisations appear to be associated with influenza A, although this may reflect the greater frequency of influenza A infections in the community. While the 2009 pandemic year had a lower all-age hospitalisation rate compared with seasonal influenza, the rate in 15–64 year-olds was higher than the seasonal influenza average, although this rate was not unusual compared with some individual seasonal influenza years. Collinearity between influenza A and B time series presented a challenge in our modelling approach, in discriminating the type-specific impact of influenza, so future modelling should use time series that include a testing denominator to limit the effect of changing testing frequency over time. Ongoing estimation of influenza burden is required to assess the burden of influenza and to verify the performance of influenza vaccination policy in reducing influenza-attributable morbidity and mortality.

## Supporting information

S1 Fig Estimated, all-age average annual rates of influenza-attributable hospitalisations, by diagnosis category using influenza A and B model. (PDF)

S2 Fig Estimated, all-age average annual rates of influenza-attributable hospitalisations, by diagnosis category using the total influenza model. (PDF)

S3 Fig Autocorrelation plots for the GAM model with 4, 6 and 8 knots per year and for the harmonic regression model, for all-age respiratory hospitalisation rates/100,000 population, for influenza A and B model. (PDF)

S1 Table Descriptive summary of average weekly hospitalisation rate per 100,000 population by principal diagnosis and age group, Australia, 2001–2013. (PDF)

S2 Table Descriptive statistics of weekly counts of influenza notifications, by influenza type and year, Australia, 2001–2013. (PDF)

S3 Table Estimated annual influenza-attributable hospitalisation ratea by influenza type, principal diagnosis, age group and year, Australia, 2001–2013. (PDF)

S4 Table Estimated annual influenza-attributable hospitalisation counts by influenza type, principal diagnosis, age group and year, Australia, 2001–2013. (PDF)

S5 Table Sensitivity analyses comparing the generalised additive model (GAM) with a 6 knot per year smoother (original model) with (A) ordinary linear regression model with a seasonal harmonic (sinusoidal model) and with (B) a GAM model with a 4 or 8 knot per year smoother. Results are for the average annual all-age estimated influenza-attributable respiratory hospitalisation rate per 100,000, Australia, 2001–2013 excluding 2009.
(PDF)

S1 File (PDF)

S2 File (PDF)
