# Supplementary material for: Estimated hospitalisations attributable to seasonal and pandemic influenza in Australia: 2001- 2013
Source: PLoS One. 2020 Apr 13;15(4):e0230705. doi: 10.1371/journal.pone.0230705 (PMC7153886; doi:10.1371/journal.pone.0230705)

**Figure S1.** Estimated, all-age average annual rates of influenza-attributable hospitalisations, by diagnosis category using influenza A and B model

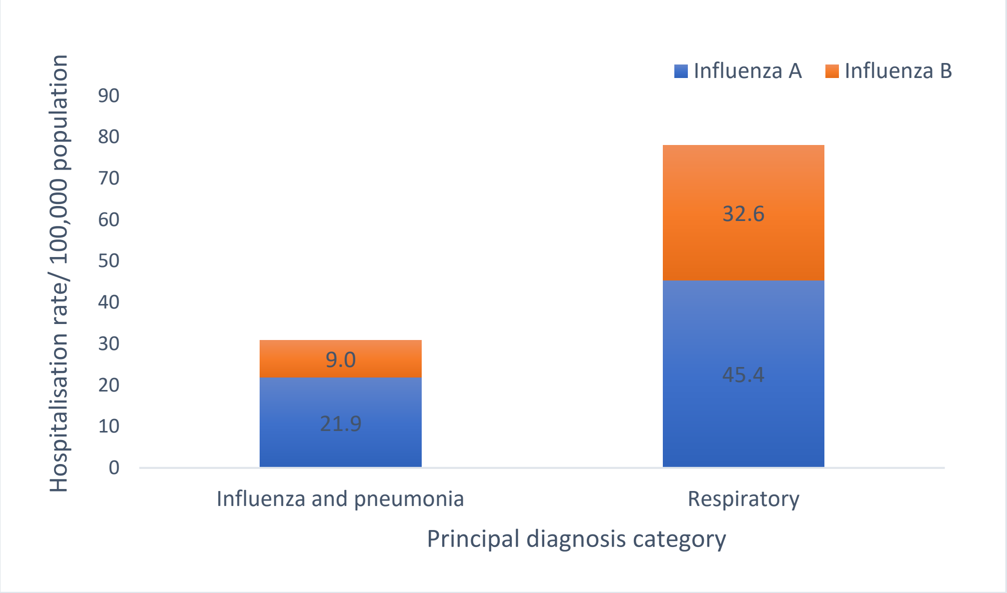

Supplement: S1 Fig — (PDF) [file pone.0230705.s001.pdf]
