# Supplementary material for: Estimated hospitalisations attributable to seasonal and pandemic influenza in Australia: 2001- 2013
Source: PLoS One. 2020 Apr 13;15(4):e0230705. doi: 10.1371/journal.pone.0230705 (PMC7153886; doi:10.1371/journal.pone.0230705)

Figure S3. Autocorrelation plots for the GAM model with 4, 6 and 8 knots per year and for the harmonic regression model, for all-age respiratory hospitalisation rates/100,000 population, for influenza A and B model

4 knots/year

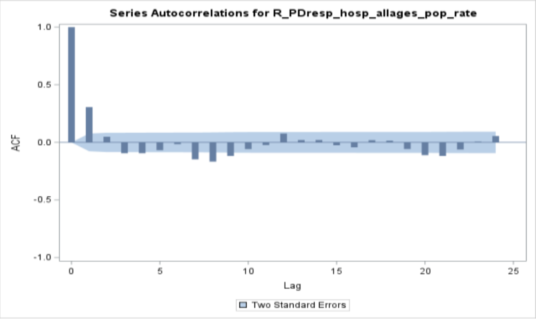

6 knots/year

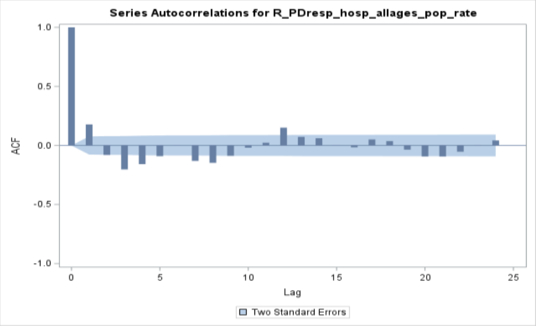

8 knots/year

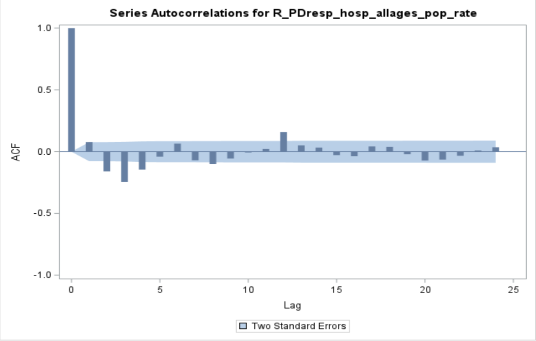

Harmonic ordinary linear regression

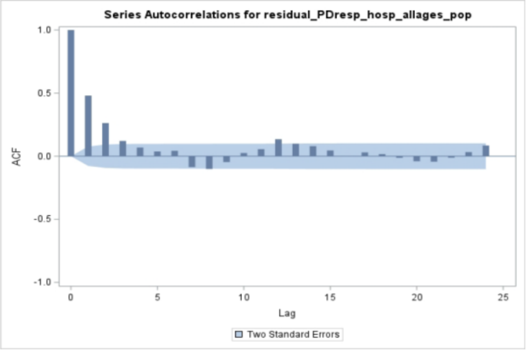

Supplement: S3 Fig — (PDF) [file pone.0230705.s003.pdf]
