# Supplementary material for: Estimated hospitalisations attributable to seasonal and pandemic influenza in Australia: 2001- 2013
Source: PLoS One. 2020 Apr 13;15(4):e0230705. doi: 10.1371/journal.pone.0230705 (PMC7153886; doi:10.1371/journal.pone.0230705)
Supplement: S1 Table — (PDF) [file pone.0230705.s004.pdf]

Table S1. Descriptive summary of average weekly hospitalisation rate per 100,000 population by principal diagnosis and age group, Australia, 2001-2013

Principal Diagnosis	Age group (years)				
	**0-14**	**15-64**	**65-84**	**≥85**	**All-age**
Influenza and pneumonia	5.4	3.1	19.8	64.9	6.5
Respiratory	47.3	17.9	75.5	162.2	32.7
