# Supplementary material for: Estimated hospitalisations attributable to seasonal and pandemic influenza in Australia: 2001- 2013
Source: PLoS One. 2020 Apr 13;15(4):e0230705. doi: 10.1371/journal.pone.0230705 (PMC7153886; doi:10.1371/journal.pone.0230705)
Supplement: S2 Table — (PDF) [file pone.0230705.s005.pdf]

Table S2. Descriptive statistics of weekly counts of influenza notifications, by influenza type and year, Australia, 2001-2013

Year	Number of notifications (N)	Mean	Medium	Minimum	Maximum	Interquartile range
Influenza A						
2001 (Jul-Dec)	940	36	21	0	119	53
2002	2681	52	12	0	254	53
2003	3129	60	8	1	424	18
2004	1575	30	12	2	149	35
2005	3408	64	27	4	238	62
2006	2343	45	20	5	219	44
2007	9233	178	26	7	1303	99
2008	4029	77	39	11	350	68
2009	58411	1123	83	19	8687	1286
2010	11976	230	97	25	1205	219
2011	19895	375	195	51	1491	336
2012	33908	652	110	38	3016	660
2013	17731	341	195	86	1336	258
**All years**	**169,259**	**260**	**50**	**0**	**8687**	**155**
Influenza B						
2001 (Jul-Dec)	111	4	5	0	9	5
2002	868	17	5	0	78	26
2003	124	2	2	0	8	2
2004	370	7	3	0	32	8
2005	1000	19	9	2	84	21
2006	877	17	7	0	72	24
2007	956	18	10	0	73	25
2008	5029	97	21	5	681	68
2009	478	9	7	0	31	9
2010	1282	25	8	0	113	38
2011	7328	138	35	7	641	194
2012	10538	203	119	11	792	257
2013	10410	200	88	17	1015	215
**All years**	**39,371**	**60**	**12**	**0**	**1015**	**36**
