# Supplementary material for: Estimated hospitalisations attributable to seasonal and pandemic influenza in Australia: 2001- 2013
Source: PLoS One. 2020 Apr 13;15(4):e0230705. doi: 10.1371/journal.pone.0230705 (PMC7153886; doi:10.1371/journal.pone.0230705)
Supplement: S3 Table — (PDF) [file pone.0230705.s006.pdf]

Table S3. Estimated annual influenza-attributable hospitalisation rate[a] by influenza type, principal diagnosis, age group and year, Australia, 2001-2013.

		**Hospitalization rate – attributable to Influenza A (95%CI)**				
Principal Diagnosis	Year	Age group				
		0-14 years	15-64 years	65-84 years	≥85 years	All-ages
Influenza and Pneumonia	2001 (Jul-Dec)	**19.5 (12.5, 26.5)**	**14.1 (11.8, 16.5)**	**17.1 (2.5, 31.7)**	**98.5 (35.2, 161.8)**	**16.7 (13.1, 20.3)**
	2002	**36.9 (29.7, 44.1)**	**9.9 (7.5, 12.3)**	**61.4 (46.3, 76.5)**	**281.7 (216.2, 347.2)**	**25.0 (21.3, 28.7)**
	2003	**68.7 (63.7, 73.8)**	**20.6 (18.9, 22.2)**	**109.3 (98.7, 119.8)**	**374.7 (328.9, 420.4)**	**45.3 (42.7, 47.9)**
	2004	**37.5 (21.8, 53.2)**	**17.5 (12.3, 22.6)**	**88.6 (55.9, 121.4)**	**336.9 (194.8, 479.0)**	**34.2 (26.2, 42.2)**
	2005	-0.8 (-15.4, 13.8)	**18.5 (13.7, 23.4)**	**32.8 (2.1, 63.4)**	**462.1 (329.4, 594.8)**	**23.0 (15.5, 30.5)**
	2006	3.5 (-16.3, 23.3)	**7.3 (0.7, 13.8)**	38.8 (-2.6, 80.3)	-54.8 (-234.3, 124.8)	9.2 (-0.9, 19.4)
	2007	**31.2 (24.7, 37.7)**	**15.5 (13.4, 17.7)**	**46.7 (33.0, 60.3)**	**253.0 (193.7, 312.3)**	**26.0 (22.7, 29.4)**
	2008	-11.6 (-29.3, 6.1)	*-17.5 (-23.3, -11.6)*	36.2 (-0.8, 73.1)	-97.1 (-257.4, 63.2)	*-11.4 (-20.5, -2.4)*
	2009	**42.2 (36.8, 47.5)**	**37.6 (35.8, 39.4)**	**34.5 (23.3, 45.6)**	**110.8 (62.4, 159.3)**	**39.4 (36.6, 42.1)**
	2010	**27.2 (14.9, 39.6)**	**20.9 (16.9, 25.0)**	13.4 (-12.4, 39.2)	-51.4 (-163.2, 60.4)	**20.0 (13.7, 26.3)**
	2011	*-29.8 (-50.3, -9.4)*	**11.4 (4.7, 18.2)**	**52.5 (9.7, 95.3)**	153.0 (-32.7, 338.6)	11.1 (0.6, 21.6)
	2012	**18.6 (9.8, 27.5)**	**19.6 (16.7, 22.5)**	**86.6 (68.0, 105.1)**	**442.2 (361.8, 522.5)**	**35.5 (31.0, 40.0)**
	2013	-15.0 (-52.8, 22.7)	**18.1 (5.6, 30.5)**	**118.3 (39.4, 197.3)**	246.0 (-96.5, 588.4)	**28.0 (8.7, 47.3)**
Respiratory	2001 (Jul-Dec)	36.1 (-9.9, 82.1)	**35.5 (26.3, 44.8)**	**55.5 (20.4, 90.7)**	**173.6 (58.1, 289.0)**	**39.7 (24.7, 54.7)**
	2002	**114.2 (66.6, 161.8)**	**18.0 (8.4, 27.5)**	**208.5 (172.2, 244.9)**	**585.5 (466.2, 704.9)**	**66.8 (51.2, 82.3)**
	2003	**129.9 (96.6, 163.1)**	**51.2 (44.5, 57.8)**	**347.9 (322.5, 373.3)**	**850.2 (766.8, 933.6)**	**111.8 (101.0, 122.7)**
	2004	88.1 (-15.3, 191.4)	**56.4 (35.7, 77.1)**	**260.5 (181.5, 339.4)**	**481.2 (222.0, 740.4)**	**92.2 (58.4, 125.9)**
	2005	-16.5 (-113.0, 80.0)	**36.3 (17.0, 55.7)**	**222.0 (148.3, 295.7)**	**896.3 (654.2, 1138.3)**	**60.1 (28.5, 91.6)**
	2006	42.3 (-88.3, 172.8)	21.5 (-4.7, 47.7)	**109.0 (9.3, 208.8)**	154.0 (-173.4, 481.5)	37.8 (-4.8, 80.5)
	2007	**69.4 (26.3, 112.5)**	**35.4 (26.7, 44.0)**	**137.9 (105.0, 170.8)**	**372.4 (264.3, 480.5)**	**59.3 (45.2, 73.3)**
	2008	-89.3 (-205.9, 27.2)	*-23.9 (-47.3, -0.5)*	**92.1 (3.0, 181.1)**	-25.6 (-318.0, 266.7)	-23.3 (-61.4, 14.7)
	2009	19.7 (-15.6, 54.9)	**60.5 (53.4, 67.5)**	**74.1 (47.1, 101.0)**	**301.1 (212.8, 389.5)**	**58.3 (46.8, 69.8)**
	2010	-16.3 (-97.6, 65.0)	**31.0 (14.7, 47.3)**	15.9 (-46.2, 78.0)	-92.8 (-296.7, 111.1)	18.3 (-8.2, 44.9)
	2011	-88.5 (-223.5, 46.5)	-14.6 (-41.7, 12.5)	92.0 (-11.1, 195.1)	**403.9 (65.2, 742.5)**	-8.2 (-52.3, 35.8)
	2012	-39.8 (-98.2, 18.6)	**37.8 (26.1, 49.5)**	**266.9 (222.3, 311.5)**	**866.2 (719.7, 1012.6)**	**66.8 (47.7, 85.8)**
	2013	-79.7 (-328.6, 169.3)	25.6 (-24.3, 75.6)	**210.4 (20.3, 400.6)**	-103.3 (-727.8, 521.2)	23.6 (-57.7, 104.9)
		**Hospitalization rate – attributable to Influenza B (95%CI)**				
Principal Diagnosis	Year	Age group				
		0-14 years	15-64 years	65-84 years	≥85 years	All-ages

Influenza and Pneumonia	2001 (Jul-Dec)	4.7 (-3.9, 13.4)	0.8 (-2.1, 3.6)	**22.9 (4.8, 40.9)**	**114.1 (35.8, 192.4)**	**5.7 (1.3, 10.1)**
	2002	**25.3 (17.7, 33.0)**	**8.3 (5.7, 10.8)**	1.1 (-15.0, 17.2)	**119.2 (49.5, 188.9)**	**12.5 (8.6, 16.4)**
	2003	-4.0 (-13.1, 5.2)	2.5 (-0.5, 5.6)	1.6 (-17.6, 20.7)	*-109.4 (-192.4, -26.5)*	-0.5 (-5.1, 4.2)
	2004	5.5 (-11.4, 22.4)	1.6 (-4.0, 7.2)	33.7 (-1.7, 69.1)	-98.8 (-252.3, 54.7)	4.6 (-4.0, 13.3)
	2005	**27.6 (12.7, 42.6)**	**6.1 (1.2, 11.1)**	**46.2 (14.9, 77.4)**	-108.7 (-244.2, 26.7)	**13.2 (5.6, 20.9)**
	2006	**39.1 (18.8, 59.3)**	**9.0 (2.3, 15.6)**	-2.4 (-44.7, 39.9)	**265.4 (82.0, 448.9)**	**17.5 (7.2, 27.9)**
	2007	**23.0 (13.5, 32.4)**	-1.4 (-4.5, 1.8)	-14.0 (-33.8, 5.8)	*-209.7 (-295.4, -124.0)*	-1.5 (-6.3, 3.4)
	2008	**32.1 (19.2, 45.0)**	**22.8 (18.5, 27.1)**	**37.4 (10.4, 64.5)**	**190.0 (72.8, 307.3)**	**29.1 (22.4, 35.7)**
	2009	*-9.6 (-18.4, -0.8)*	*-14.0 (-16.9, -11.1)*	**21.9 (3.6, 40.3)**	**107.7 (28.0, 187.3)**	*-6.9 (-11.4, -2.4)*
	2010	-1.8 (-15.5, 11.9)	*-7.0 (-11.5, -2.5)*	23.0 (-5.6, 51.6)	**214.1 (90.1, 338.1)**	1.4 (-5.6, 8.4)
	2011	**45.5 (28.3, 62.7)**	1.4 (-4.3, 7.1)	*-38.6 (-74.6, -2.6)*	*-214.3 (-370.3, -58.3)*	1.0 (-7.8, 9.8)
	2012	**18.1 (7.2, 29.1)**	**7.4 (3.8, 11.1)**	**32.7 (9.8, 55.7)**	**174.5 (75.1, 274.0)**	**15.7 (10.0, 21.3)**
	2013	**43.4 (12.0, 74.7)**	10.2 (-0.2, 20.6)	-43.8 (-109.4, 21.8)	-53.2 (-337.7, 231.2)	9.0 (-7.1, 25.0)
Respiratory	2001 (Jul-Dec)	21.5 (-35.5, 78.4)	-5.4 (-16.8, 6.0)	**98.4 (54.9, 141.9)**	**156.8 (14.0, 299.6)**	14.3 (-4.3, 32.9)
	2002	**86.9 (36.2, 137. 6)**	**28.8 (18.7, 39.0)**	**44.9 (6.2, 83.7)**	**289.0 (161.8, 416.1)**	**46.1 (29.6, 62.7)**
	2003	34.2 (-26.1, 94.6)	**17.7 (5.6, 29.8)**	-1.3 (-47.4, 44.8)	-27.6 (-178.9, 123.7)	18.4 (-1.3, 38.1)
	2004	-16.3 (-127.9, 95.2)	-15.1 (-37.4, 7.3)	42.9 (-42.4, 128.1)	-28.2 (-308.0, 251.7)	-8.7 (-45.1, 27.7)
	2005	64.3 (-34.2, 162.8)	19.5 (-0.3, 39.2)	**70.7 (-4.6, 145.9)**	-135.3 (-382.3, 111.8)	31.8 (-0.4, 63.9)
	2006	60.4 (-70.3, 193.8)	22.0 (-4.7, 48.8)	101.7 (-0.2, 203.7)	**381.1 (46.4, 715.7)**	**44.1 (0.6, 87.7)**
	2007	**122.7 (60.4, 185.0)**	0.2 (-12.3, 12.7)	-44.7 (-92.3, 2.9)	*-168.2 (-324.5, -11.9)*	16.1 (-4.2, 36.4)
	2008	**94.5 (9.3, 179.7)**	**52.9 (35.8, 70.0)**	**138.9 (73.8, 204.0)**	**432.9 (219.2, 646.7)**	**77.3 (49.5, 105.1)**
	2009	-46.6 (-104.5, 11.3)	*-31.0 (-42.7, -19.4)*	**46.0 (1.8, 90.3)**	**158.9 (13.6, 304.2)**	*-21.8 (-40.7, -2.9)*
	2010	**103.2 (13.0, 193.3)**	-8.1 (-26.1, 10.0)	4.8 (-64.0, 73.7)	**239.8 (13.7, 466.0)**	18.4 (-11.0, 47.9)
	2011	**153.4 (40.0, 266.8)**	**28.1 (5.3, 50.8)**	-22.1 (-108.7, 64.6)	*-375.1 (-659.6, -90.6)*	**38.5 (1.5, 75.6)**
	2012	51.4 (-20.9, 123.7)	**17.7 (3.2, 32.2)**	**123.3 (68.1, 178.5)**	**233.0 (51.6, 414.4)**	**40.9 (17.3, 64.5)**
	2013	140.0 (-66.8, 346.8)	30.1 (-11.3, 71.6)	-16.3 (-174.3, 141.7)	381.6 (-137.2, 900.4)	53.8 (-13.8, 121.3)
		**Hospitalization rate – attributable to total influenza (95%CI)**				
Principal Diagnosis	Year	Age group				
		0-14 years	15-64 years	65-84 years	≥85 years	All-ages
Influenza and Pneumonia	2001 (Jul-Dec)	**24.3 (19.5, 29.1)**	**17.4 (15.8, 19.0)**	**28.9 (19.1, 38.7)**	**148.0 (104.6, 191.4)**	**21.9 (19.5, 24.3)**
	2002	**54.4 (48.9, 59.9)**	**15.1 (13.3, 17.0)**	**75.7 (64.4, 87.0)**	**386.5 (336.4, 436.6)**	**35.1 (32.3, 37.9)**
	2003	**71.4 (67.4, 75.5)**	**22.2 (20.9, 23.6)**	**117.5 (109.1, 125.9)**	**370.7 (333.6, 407.9)**	**47.8 (45.8, 49.9)**
	2004	**43.5 (38.0, 49.0)**	**19.3 (17.5, 21.1)**	**117.8 (106.5, 129.2)**	**267.1 (216.9, 317.4)**	**39.0 (36.2, 41.8)**
	2005	**21.0 (15.0, 26.9)**	**23.8 (21.8, 25.7)**	**70.3 (58.1, 82.6)**	**374.5 (320.5, 428.5)**	**33.8 (30.8, 36.8)**
	2006	**38.3 (32.7, 44.0)**	**15.7 (13.8, 17.5)**	**39.7 (28.1, 51.4)**	**165.7 (114.2, 217.2)**	**25.2 (22.3, 28.1)**

	2007	**39.7 (35.5, 44.0)**	**16.3 (14.9, 17.7)**	**46.0 (37.2, 54.8)**	**215.9 (177.0, 254.9)**	**27.5 (25.4, 29.7)**
	2008	**34.2 (29.3, 39.2)**	**18.4 (16.7, 20.0)**	**71.9 (61.8, 82.0)**	**185.2 (140.5, 229.9)**	**30.4 (27.9, 32.9)**
	2009	**41.4 (37.3, 45.5)**	**36.2 (34.8, 37.6)**	**37.5 (29.0, 46.1)**	**125.5 (87.8, 163.2)**	**38.9 (36.8, 41.0)**
	2010	**27.9 (22.6, 33.3)**	**18.2 (16.4, 20.0)**	**26.6 (15.6, 37.6)**	**62.5 (13.9, 111.1)**	**21.8 (19.1, 24.5)**
	2011	**21.8 (15.5, 28.2)**	**13.1 (11.0, 15.2)**	7.5 (-5.6, 20.6)	*-96.6 (-154.4, -38.8)*	**12.1 (8.9, 15.3)**
	2012	**30.3 (25.0, 35.6)**	**26.4 (24.6, 28.1)**	**116.5 (105.5, 127.5)**	**597.8 (549.2, 646.4)**	**48.8 (46.1, 51.5)**
	2013	**38.3 (31.7, 44.9)**	**28.2 (26.0, 30.4)**	**52.3 (38.8, 65.8)**	**161.6 (101.8, 221.4)**	**35.6 (32.3, 39.0)**
Respiratory	2001 (Jul-Dec)	**48.2 (17.2, 79.3)**	**41.7 (35.4, 48.0)**	**97.8 (74.2, 121.3)**	**270.9 (193.0, 348.9)**	**52.4 (42.3, 62.5)**
	2002	**169.3 (133.4, 205.1)**	**32.5 (25.3, 39.7)**	**269.9 (242.7, 297.2)**	**825.3 (735.3, 915.2)**	**98.0 (86.3, 109.7)**
	2003	**139.1 (112.6, 165.7)**	**57.1 (51.8, 62.5)**	**369.6 (349.4, 389.8)**	**888.7 (822.0, 955.4)**	**120.7 (112.1, 129.4)**
	2004	**73.7 (37.8, 109.7)**	**45.1 (37.8, 52.3)**	**300.3 (273.0, 327.5)**	**475.3 (385.1, 565.5)**	**86.3 (74.6, 98.0)**
	2005	29.6 (-9.1, 68.2)	**53.8 (46.0, 61.6)**	**284.8 (255.5, 314.2)**	**783.3 (686.3, 880.3)**	**86.3 (73.7, 98.9)**
	2006	**100.7 (63.9, 137.6)**	**42.9 (35.5, 50.3)**	**207.9 (179.9, 235.9)**	**484.7 (392.2, 577.2)**	**80.1 (68.1, 92.1)**
	2007	**108.9 (81.0, 136.7)**	**37.9 (32.3, 43.5)**	**134.1 (112.9, 155.2)**	**351.1 (281.2, 421.1)**	**67.9 (58.8, 76.9)**
	2008	**65.1 (33.1, 97.1)**	**53.3 (46.8, 59.7)**	**240.1 (215.8, 264.4)**	**546.1 (465.8, 626.4)**	**85.4 (75.0, 95.8)**
	2009	14.1 (-12.9, 41.1)	**57.2 (51.7, 62.6)**	**80.5 (60.0, 101.0)**	**323.9 (256.2, 391.6)**	**56.1 (47.4, 64.9)**
	2010	**40.5 (5.8, 75.3)**	**28.4 (21.4, 35.4)**	19.2 (-7.1, 45.6)	31.5 (-55.8, 118.7)	**29.7 (18.3, 41.0)**
	2011	**78.6 (37.2, 120.0)**	**17.7 (9.4, 26.0)**	**64.9 (33.5, 96.3)**	-31.2 (-135.1, 72.6)	**34.1 (20.6, 47.5)**
	2012	-22.2 (-57.0, 12.5)	**52.4 (45.4, 59.4)**	**369.7 (343.3, 396.1)**	**1120.0 (1032.7, 1207.2)**	**97.2 (85.9, 108.5)**
	2013	**91.3 (48.5, 134.1)**	**58.9 (50.2, 67.5)**	**168.1 (135.6, 200.6)**	**377.8 (270.3, 485.2)**	**84.6 (70.7, 98.6)**

[a] Rate per 100,000 population; Positive statistically significant estimates are shown in bold and negative statistically significant estimates are presented by italics.
