# Supplementary material for: Estimated hospitalisations attributable to seasonal and pandemic influenza in Australia: 2001- 2013
Source: PLoS One. 2020 Apr 13;15(4):e0230705. doi: 10.1371/journal.pone.0230705 (PMC7153886; doi:10.1371/journal.pone.0230705)
Supplement: S4 Table — (PDF) [file pone.0230705.s007.pdf]

Table S4. Estimated annual influenza-attributable hospitalisation counts by influenza type, principal diagnosis, age group and year, Australia, 2001-2013.

		**Number of hospitalizations – attributable to Influenza A (95%CI)**				
Principal Diagnosis	Year	Age group				
		0-14 years	15-64 years	65-84 years	≥85 years	All-ages
Influenza and Pneumonia	2001 (Jul-Dec)	**771 (495, 1,048)**	**1,828 (1,529, 2,126)**	**370 (54, 686)**	**261 (93, 429)**	**3,227 (2,537, 3,917)**
	2002	**1463 (1177, 1749)**	**1299 (986, 1611)**	**1349 (1017, 1680)**	**769 (591, 948)**	**4,876 (4,155, 5,597)**
	2003	**2729 (2529, 2929)**	**2729 (2507, 2950)**	**2446 (2210, 2682)**	**1,051 (923, 1,179)**	**8,951 (8,441, 9,461)**
	2004	**1492 (868, 2115)**	**2346 (1649, 3042)**	**2021 (1273, 2768)**	**976 (564, 1388)**	**6,837 (5,234, 8,440)**
	2005	-32 (-615, 551)	**2,517 (1,858, 3,175)**	**757 (50, 1,465)**	**1,401 (999, 1,804)**	**4,642 (3,129, 6,155)**
	2006	142 (-652, 935)	**1005 (100, 1910)**	914 (-60, 1888)	-176 (-752, 400)	1894 (-183, 3971)
	2007	**1,264 (999, 1,529)**	**2,187 (1,883, 2,492)**	**1,122 (793, 1,451)**	**857 (656, 1,058)**	**5,430 (4,732, 6,128)**
	2008	-476 (-1202, 251)	*-2514 (-3356, -1673)*	891 (-20, 1801)	-345 (-913, 224)	*-2,439 (-4,367, -510)*
	2009	**1,755 (1,533, 1,977)**	**5510 (5251, 5769)**	**871 (589, 1153)**	**409 (230, 588)**	**8,544 (7,950, 9,138)**
	2010	**1,145 (626, 1,663)**	**3,114 (2,508, 3,721)**	350 (-325, 1,025)	-200 (-636, 235)	**4,419 (3,025, 5,813)**
	2011	*-1,265 (-2,133, -397)*	**1,721 (703, 2,739)**	**1,414 (261, 2,567)**	619 (-132, 1,370)	**2,488 (144, 4,832)**
	2012	**802 (421, 1,183)**	**2,984 (2,538, 3,430)**	**2,428 (1,908, 2,947)**	**1,864 (1,525, 2,202)**	**8,078 (7,047, 9,110)**
	2013	-657 (-2,310, 995)	**2,788 (860, 4,717)**	**3,446 (1,146, 5,746)**	1,082 (-424, 2,588)	**6,492 (2,014, 10,970)**
Respiratory	2001 (Jul-Dec)	1,430 (-393, 3,253)	**4,593 (3,400, 5,786)**	**1,200 (440, 1,961)**	**460 (154, 766)**	**7,667 (4,765, 10,569)**
	2002	**4,528 (2,641, 6,415)**	**2,351 (1,103, 3,600)**	**4,580 (3,782, 5,379)**	**1,599 (1,273, 1,925)**	**13,034 (10,001, 16,066)**
	2003	**5,156 (3,836, 6,475)**	**6,787 (5,902, 7,671)**	**7,785 (7,216, 8,353)**	**2,384 (2,151, 2,618)**	**22,091 (19,947, 24,235)**
	2004	3,501 (-607, 7,610)	**7,579 (4,794, 10,364)**	**5,938 (4,139, 7,738)**	**1,394 (643, 2,145)**	**18,417 (11,675, 25,159)**
	2005	-656 (-4,501, 3,188)	**4,941 (2,310, 7,572)**	**5,132 (3,429, 6,836)**	**2,718 (1,984, 3,452)**	**12,128 (5,765, 18,490)**
	2006	1,695 (-3,538, 6,927)	2,974 (-643, 6,592)	**2,565 (219, 4,911)**	494 (-557, 1,546)	7,759 (-978, 16,496)
	2007	**2,813 (1,066, 4,559)**	**4,980 (3,764, 6,197)**	**3,316 (2,524, 4,108)**	**1,261 (895, 1,627)**	**12,370 (9,433, 15,307)**
	2008	-3,671 (-8,461, 1,118)	*-3,439 (-6,802, -76)*	**2,268 (75, 4,461)**	-91 (-1,129, 947)	-4,970 (-13,082, 3,142)
	2009	819 (-647, 2,284)	**8859 (7824, 9894)**	**1,871 (1,191, 2,551)**	**1,112 (785, 1,438)**	**12,654 (10,157, 15,151)**
	2010	-686 (-4,105, 2,732)	**4,611 (2,185, 7,037)**	417 (-1,207, 2,042)	-362 (-1,157, 433)	4,047 (-1,816, 9,910)
	2011	-3,752 (-9,475, 1,971)	-2194 (-6264, 1876)	2,477 (-300, 5,254)	**1,634 (264, 3,004)**	-1,841(-11,701, 8,018)
	2012	-1,714 (-4,228, 800)	**5,755 (3,972, 7,538)**	**7,486 (6,235, 8,737)**	**3,651 (3,034, 4,268)**	**15,193 (10,855, 19,532)**
	2013	-3,488 (-14,383, 7407)	4,959 (-3,752, 11,669)	**6,129 (590, 11,667)**	-454 (-3,201, 2,292)	5,475 (-13,359, 24,309)
		**Number of hospitalizations – attributable to Influenza B (95%CI)**				
Principal Diagnosis	Year	Age group				
		0-14 years	15-64 years	65-84 years	≥85 years	All-ages

Influenza and Pneumonia	2001 (Jul-Dec)	188 (-154, 530)	102 (-267, 472)	**495 (105, 886)**	**303 (95, 511)**	**1,101 (247, 1,955)**
	2002	**1,005 (700, 1,310)**	**1,080 (748, 1,413)**	24 (-329, 377)	**325 (135, 515)**	**2,435 (1,667, 3,202)**
	2003	-159 (-522, 205)	337 (-64, 738)	35 (-393, 463)	*-307 (-539, -74)*	-90 (-1,014, 834)
	2004	220 (-453, 893)	213 (-539, 965)	768 (-38, 1575)	-286 (-730, 158)	924 (-806, 2,654)
	2005	**1,101 (506, 1,696)**	**833 (161, 1,505)**	1,067 (345, 1,790)	-330 (-742, 81)	**2,670 (1,126, 4,215)**
	2006	**1,566 (755, 2,377)**	**1,236 (312, 2,161)**	-56 (-1,050, 939)	**850 (263, 1,438)**	**3,588 (1,467, 5,710)**
	2007	**932 (548, 1,315)**	-194 (-634, 247)	-337 (-813, 139)	*-712 (-1,003, -421)*	-306 (-1,317, 704)
	2008	**1,320 (788, 1,851)**	**3,281 (2,666, 3,896)**	922 (256, 1,588)	**675 (259, 1,091)**	**6,196 (4,785, 7,606)**
	2009	*-399 (-764, -34)*	*-2,052 (-2,476, -1,627)*	**553 (90, 1,015)**	**396 (103, 688)**	*-1,502 (-2,476, -529)*
	2010	-76 (-652, 499)	-1039 (-1712, -365)	603 (-146, 1352)	**836 (352, 1320)**	300 (-1,247, 1,847)
	2011	**1,929 (1,199, 2,658)**	216 (-639, 1,071)	-1040 (-2009, -70)	*-868 (-1499, -236)*	234 (-1,735, 2,204)
	2012	**781 (308, 1,254)**	**1,131 (578, 1,684)**	**921 (276, 1,565)**	**737 (317, 1158)**	**3,566 (2,287, 4,845)**
	2013	**1,899 (525, 3,272)**	1,574 (-29, 3,176)	-1,277 (-3,189, 635)	-234 (-1487, 1,018)	2,080 (-1,642, 5,801)
Respiratory	2001 (Jul-Dec)	850 (-1,405, 3105)	-702 (-2,178, 775)	**2,129 (1,188, 3,070)**	**416 (37, 795)**	2,763 (-829, 6,354)
	2002	**3,445 (1,436, 5,455)**	**3,767 (2,439, 5,096)**	986 (136, 1,836)	**788 (441, 1,134)**	**8994 (5,766, 12,222)**
	2003	1,360 (-1,035, 3,754)	**2,351 (747, 3,954)**	-29 (-1,060, 1,001)	-77 (-501, 347)	3637 (-251, 7,525)
	2004	-649 (-5,085, 3,786)	-2,024 (-5,030, 981)	977 (-965, 2,919)	-81 (-891, 728)	-1740 (-9,016, 5,537)
	2005	2,563 (-1,362, 6,487)	2,651 (-35, 5,337)	1,635 (-105, 3,374)	-411 (-1,161, 339)	6,419 (-77, 12,915)
	2006	2,422 (-2,923, 7,767)	3,040 (-655, 6,735)	2,392 (-4, 4,787)	**1,221 (149, 2,293)**	**9,040 (117, 17,963)**
	2007	**4,976 (2,450, 7,503)**	26 (-1,735, 1,787)	*-1,076 (-2,222, 69)*	*-571 (-1102, -40)*	3,363 (-888, 7,613)
	2008	**3,883 (381, 7,386)**	**7,614 (5,155, 10,074)**	**3,423 (1,819, 5,027)**	**1,537 (778, 2,296),**	**16,480 (10,548, 22,412)**
	2009	-1937 (-4,342, 469)	*-4537 (-6,235, -2,838)*	**1,160 (45, 2,274)**	**584 (50, 1,118)**	*-4718 (-8,814, -622)*
	2010	**4,341 (548, 8,133)**	-1,200 (-3,891, 1,492)	126 (-1,678, 1,930)	**936 (53, 1,819)**	4,071 (-2,435, 10,577)
	2011	**6,506 (1,698, 11,313)**	**4,220 (800, 7,640)**	-595 (-2,929, 1,740)	*-1,519 (-2,671, -367)*	**8,621 (337, 16,905)**
	2012	2,216 (-900, 5,333)	**2,690 (480, 4,901)**	**3,466 (1,913, 5,019)**	**984 (218, 1,751)**	**9,317 (3,938, 14,696)**
	2013	6,131 (-2,924, 15,185),	4,657 (-1,750, 11,065)	-476 (-5,081, 4,130)	1,680 (-604, 3,964)	12,463 (-3,190, 28,116)
		**Number of hospitalizations – attributable to total influenza (95%CI)**				
Principal Diagnosis	Year	Age group				
		0-14 years	15-64 years	65-84 years	≥85 years	All-ages
Influenza and Pneumonia	2001 (Jul-Dec)	**962 (773, 1,151)**	**2,249 (2,043, 2,454)**	**624 (412, 836)**	**392 (277, 507)**	**4,227 (3,761, 4,692)**
	2002	**2,157 (1,938, 2,375)**	**1,979 (1,740, 2,219)**	**1,663 (1,414, 1,911)**	**1,055 (918, 1,192)**	**6,850 (6,307, 67,392)**
	2003	**2,836 (2,674, 2,998)**	**2,949 (2,768, 3,129)**	**2,629 (2,441, 2,817)**	**1,040 (936, 1,144)**	**9,451 (9,043, 9,858)**
	2004	**1,729 (1,509, 1,948)**	**2,594 (2,347, 2,841)**	**2,686 (2,427, 2,945)**	**774 (628, 919)**	**7,792 (7,235, 8,348)**
	2005	**836 (600, 1,072)**	**3,231 (2,962, 3,500)**	**1,626 (1,344, 1,909)**	**1,136 (972, 1,300)**	**6,828 (6,223, 7,434)**
	2006	**1,536 (1,309, 1,762)**	**2,163 (1,902, 2,423)**	**934 (660, 1,208)**	**532 (366, 697)**	**5,166 (4,580, 5,752)**
	2007	**1,611 (1,437, 1,784)**	**2,298 (2,097, 2,498)**	**1,105 (894, 1,317)**	**731 (599, 863)**	**5,745 (5,294, 6,197)**

	2008	**1,408 (1,206, 1,609)**	**2,640 (2,405, 2,876)**	**1,772 (1,523, 2,021)**	**657 (499, 816)**	**6,478 (5,949, 7,007)**
	2009	**1,722 (1,550, 1,894)**	**5,306 (5,104, 5,508)**	**948 (733, 1,164)**	**463 (324, 602)**	**8,438 (7,984, 8,892)**
	2010	**1,174 (50, 1,399)**	**2,706 (2,441, 2,970)**	**695 (408, 983)**	**244 (54, 433)**	**4,817 (4,222, 5,413)**
	2011	**926 (656, 1,195)**	**1,972 (1,654, 2,290)**	202 (-151, 554)	*-391 (-625, -157)*	**2,706 (1,988, 3,424)**
	2012	**1,304 (1,075, 1,534)**	**4,013 (3,742, 4,284)**	**3,269 (2,961, 3,577)**	**2,521 (2,316, 2,726)**	**11,106 (10,492, 11,720)**
	2013	**1,677 (1,389, 1,965)**	**4,356 (4,018, 4,694)**	**1,523 (1,129, 1,918)**	**711 (448, 974)**	**8,260 (7,490, 9,030)**
Respiratory	2001 (Jul-Dec)	**1,911 (681, 3,140)**	**5,390 (4,581, 6,198)**	**2,114 (1,604, 2,624)**	**718 (512, 925)**	**10,127 (8,175, 12,080)**
	2002	**6,712 (5,291, 8,133)**	**4,253 (3,309, 5,197)**	**5,928 (5,330, 6,526)**	**2,253 (2,007, 2,499)**	**19,120 (16,844, 21,396)**
	2003	**5,524 (4,469, 6,579),**	**7,574 (6,864, 8,284),**	**8,271 (7,819, 8,723)**	**2,493 (2,306, 2,680)**	**23,847(22,139, 25,556)**
	2004	**2,932 (1,504, 4,360)**	**6,057 (5,085, 7,029)**	**6,845 (6,223, 7,467)**	**1,376 (1,115, 1,638)**	**17,243 (14,906, 19,579)**
	2005	1,178 (-361, 2,718)	**7,316 (6,258, 8,374)**	**6,585 (5,907, 7,264)**	**2,376 (2,082, 2,670)**	**17,435 (14,895, 19,976)**
	2006	**4,037 (2,561, 5,514)**	**5,925 (4,899, 6,950)**	**4,890 (4,232, 5,548)**	**1,555 (1,258, 1,852)**	**16,411 (13,952, 18,870)**
	2007	**4,413 (3,284, 5,542)**	**5,337 (4,547, 6,127)**	**3,225 (2,716, 3,734)**	**1,189 (952, 1,426)**	**14,165 (12,272, 16,058)**
	2008	**2,675 (1,360, 3,989)**	**7,665 (6,738, 8,592)**	**5,914 (5,316, 6,512)**	**1,938 (1,653, 2,223)**	**18,198 (15,979, 20,418)**
	2009	586 (-536, 1,708)	**8,374 (7,578, 9,170)**	**2,034 (1,517, 2,551)**	**1,196 (946, 1,445)**	**12,185 (10,280, 14,091)**
	2010	**1,705 (244, 3,166)**	**4,225 (3,183, 5,266)**	504 (-187, 1,194)	123 (-217, 463)	**6,552 (4,053, 9,052)**
	2011	**3,332 (1,578, 5,086)**	**2,660 (1,408, 3,913)**	**1,747 (901, 2,593)**	-126 (-547, 294)	**7,622 (4,609, 10,634)**
	2012	-957 (-2,454, 539)	**7,982 (6,916, 9,048)**	**10,375 (9,634, 11,115)**	**4,724 (4,356, 5,092)**	**22,119 (19,543, 24,694)**
	2013	**3,997 (2,123, 5,870)**	**9,090 (7,759, 10,422)**	**489 (3,950, 5,844)**	**1,662 (1,189, 2,135)**	**19,614 (16,385, 22,843)**

Positive statistically significant estimates are shown in bold and negative statistically significant estimates are presented by italics.
