# Supplementary material for: Estimated hospitalisations attributable to seasonal and pandemic influenza in Australia: 2001- 2013
Source: PLoS One. 2020 Apr 13;15(4):e0230705. doi: 10.1371/journal.pone.0230705 (PMC7153886; doi:10.1371/journal.pone.0230705)
Supplement: S5 Table — Results are for the average annual all-age estimated influenza-attributable respiratory hospitalisation rate per 100,000, Australia, 2001–2013 excluding 2009. (PDF) [file pone.0230705.s008.pdf]

Table S5. Sensitivity analyses comparing the generalised additive model (GAM) with a 6 knot per year smoother (original model) with (A) ordinary linear regression model with a seasonal harmonic (sinusoidal model) and with (B) a GAM model with a 4 or 8 knot per year smoother. Results are for the average annual all-age estimated influenza-attributable respiratory hospitalisation rate per 100,000, Australia, 2001-2013 excluding 2009.

(A) GAM model and Sinusoidal model

GAM model with 6 knots/year (original model)<br>Rate (95% CI)		Linear Regression with annual harmonic seasonal term (sinusoidal model)<br>Rate (95% CI)	
Influenza A	Influenza B	Influenza A	Influenza B
45.4 (34.9, 55.9)	32.6 (22.8, 42.4)	40.2 (26.9, 53.5)	23.4 (11.0, 35.9)

(B) GAM models with 4 and 8 splines per year

GAM model with 4 knots/year<br>Rate (95% CI)		GAM model with 8 knots/year<br>Rate (95% CI)	
Influenza A	Influenza B	Influenza A	Influenza B
46.8 (35.1, 58.5)	42.9 (31.9, 53.9)	44.8 (35.1, 54.5)	29.5 (20.4, 38.5)

CI- confidence interval.
