# Supplementary material for: Estimated hospitalisations attributable to seasonal and pandemic influenza in Australia: 2001- 2013
Source: PLoS One. 2020 Apr 13;15(4):e0230705. doi: 10.1371/journal.pone.0230705 (PMC7153886; doi:10.1371/journal.pone.0230705)

GAM model output for all respiratory hospitalisations in persons of all ages, by influenza type

The GAM Procedure
Dependent Variable: PDresp_hosp_allages_pop_rate
Regression Model Component(s): week_before_Xmas Xmas_week new_year_week 1week_after_NY 2weeks_after_NY 3weeks_after_NY week_Australia_day one_week before_easter easter_week week_other_publicHday_Azday total_A noti_var_2001 total_A noti_var_2002 total_A noti_var_2003 total_A noti_var_2004 total_A noti_var_2005 total_A noti_var_2006 total_A noti_var_2007 total_A noti_var_2008 total_A noti_var_2009 total_A noti_var_2010 total_A noti_var_2011 total_A noti_var_2012 total_A noti_var_2013 total_A noti_var_2014 total_B noti_var_2001 total_B noti_var_2002 total_B noti_var_2003 total_B noti_var_2004 total_B noti_var_2005 total_B noti_var_2006 total_B noti_var_2007 total_B noti_var_2008 total_B noti_var_2009 total_B noti_var_2010 total_B noti_var_2011 total_B noti_var_2012 total_B noti_var_2013 total_B noti_var_2014
Smoothing Model Component(s): spline(wk_number)

Summary of Input Data Set	
Number of Observations	679
Number of Missing Observations	0
Distribution	Gaussian
Link Function	Identity

Iteration Summary and Fit Statistics	
Final Number of Backfitting Iterations	218
Final Backfitting Criterion	9.291372E-16
The Deviance of the Final Estimate	882.69079209

The backfitting algorithm converged.

Regression Model Analysis
Parameter Estimates

Parameter	Parameter Estimate	Standard Error	t Value	Pr > |t|
Intercept	30.35056	0.14780	205.35	<.0001
week_before_Xmas	-1.66540	0.34239	-4.86	<.0001
Xmas_week	-7.70067	0.35495	-21.69	<.0001
new_year_week	-6.94736	0.35535	-19.55	<.0001
1week_after_NY	-4.42837	0.35489	-12.48	<.0001
2weeks_after_NY	-3.23998	0.35484	-9.13	<.0001
3weeks_after_NY	-1.70920	0.39557	-4.32	<.0001
week_Australia_day	-3.73488	0.39580	-9.44	<.0001
one_week before_easter	-1.63288	0.35491	-4.60	<.0001
easter_week	-1.61235	0.36249	-4.45	<.0001
week_other_publicHday_Azday	-2.86775	0.36227	-7.92	<.0001
total_A noti_var_2001	0.04223	0.00816	5.18	<.0001
total_A noti_var_2002	0.02491	0.00296	8.42	<.0001
total_A noti_var_2003	0.03574	0.00177	20.19	<.0001
total_A noti_var_2004	0.05852	0.01093	5.35	<.0001
total_A noti_var_2005	0.01762	0.00472	3.74	0.0002
total_A noti_var_2006	0.01615	0.00928	1.74	0.0823
total_A noti_var_2007	0.00642	0.00077751	8.26	<.0001
total_A noti_var_2008	-0.00579	0.00482	-1.20	0.2303
total_A noti_var_2009	0.00099807	0.00010048	9.93	<.0001
total_A noti_var_2010	0.00153	0.00113	1.35	0.1767
total_A noti_var_2011	-0.00041379	0.00113	-0.37	0.7145

The GAM Procedure
Dependent Variable: PDresp_hosp_allages_pop_rate
Regression Model Component(s): week_before_Xmas Xmas_week new_year_week 1week_after_NY 2weeks_after_NY 3weeks_after_NY week_Australia_day one_week before_easter easter_week week_other_publicHday_Azday total_A noti_var_2001 total_A noti_var_2002 total_A noti_var_2003 total_A noti_var_2004 total_A noti_var_2005 total_A noti_var_2006 total_A noti_var_2007 total_A noti_var_2008 total_A noti_var_2009 total_A noti_var_2010 total_A noti_var_2011 total_A noti_var_2012 total_A noti_var_2013 total_A noti_var_2014 total_B noti_var_2001 total_B noti_var_2002 total_B noti_var_2003 total_B noti_var_2004 total_B noti_var_2005 total_B noti_var_2006 total_B noti_var_2007 total_B noti_var_2008 total_B noti_var_2009 total_B noti_var_2010 total_B noti_var_2011 total_B noti_var_2012 total_B noti_var_2013 total_B noti_var_2014
Smoothing Model Component(s): spline(wk_number)

Regression Model Analysis
Parameter Estimates

Parameter	Parameter Estimate	Standard Error	t Value	Pr > |t|
total_A noti_var_2012	0.00197	0.00028687	6.86	<.0001
total_A noti_var_2013	0.00133	0.00234	0.57	0.5691
total_A noti_var_2014	-0.00125	0.00212	-0.59	0.5558
total_B noti_var_2001	0.12880	0.08543	1.51	0.1322
total_B noti_var_2002	0.05314	0.00973	5.46	<.0001
total_B noti_var_2003	0.14855	0.08103	1.83	0.0673
total_B noti_var_2004	-0.02354	0.05023	-0.47	0.6395
total_B noti_var_2005	0.03177	0.01640	1.94	0.0533
total_B noti_var_2006	0.05031	0.02534	1.99	0.0476
total_B noti_var_2007	0.01683	0.01086	1.55	0.1216
total_B noti_var_2008	0.01537	0.00282	5.45	<.0001
total_B noti_var_2009	-0.04557	0.02019	-2.26	0.0244
total_B noti_var_2010	0.01437	0.01172	1.23	0.2205
total_B noti_var_2011	0.00526	0.00258	2.04	0.0419
total_B noti_var_2012	0.00388	0.00114	3.39	0.0007
total_B noti_var_2013	0.00516	0.00331	1.56	0.1192
total_B noti_var_2014	0.01321	0.01274	1.04	0.3001
Linear(wk_number)	0.00452	0.00039745	11.37	<.0001

Smoothing Model Analysis
Fit Summary for Smoothing Components

Component	Smoothing Parameter	DF	GCV	Num Unique Obs
Spline(wk_number)	0.994167	78.000000	1.670416	679

Smoothing Model Analysis
Analysis of Deviance

Source	DF	Sum of Squares	Chi-Square	Pr > ChiSq
Spline(wk_number)	78.00000	4108.495548	2611.1817	<.0001

The GAM Procedure
Dependent Variable: PDresp_hosp_allages_pop_rate
Regression Model Component(s): week_before_Xmas Xmas_week new_year_week 1week_after_NY 2weeks_after_NY 3weeks_after_NY week_Australia_day one_week before_easter easter_week week_other_publicHday_Azday total_A noti_var_2001 total_A noti_var_2002 total_A noti_var_2003 total_A noti_var_2004 total_A noti_var_2005 total_A noti_var_2006 total_A noti_var_2007 total_A noti_var_2008 total_A noti_var_2009 total_A noti_var_2010 total_A noti_var_2011 total_A noti_var_2012 total_A noti_var_2013 total_A noti_var_2014 total_B noti_var_2001 total_B noti_var_2002 total_B noti_var_2003 total_B noti_var_2004 total_B noti_var_2005 total_B noti_var_2006 total_B noti_var_2007 total_B noti_var_2008 total_B noti_var_2009 total_B noti_var_2010 total_B noti_var_2011 total_B noti_var_2012 total_B noti_var_2013 total_B noti_var_2014
Smoothing Model Component(s): spline(wk_number)

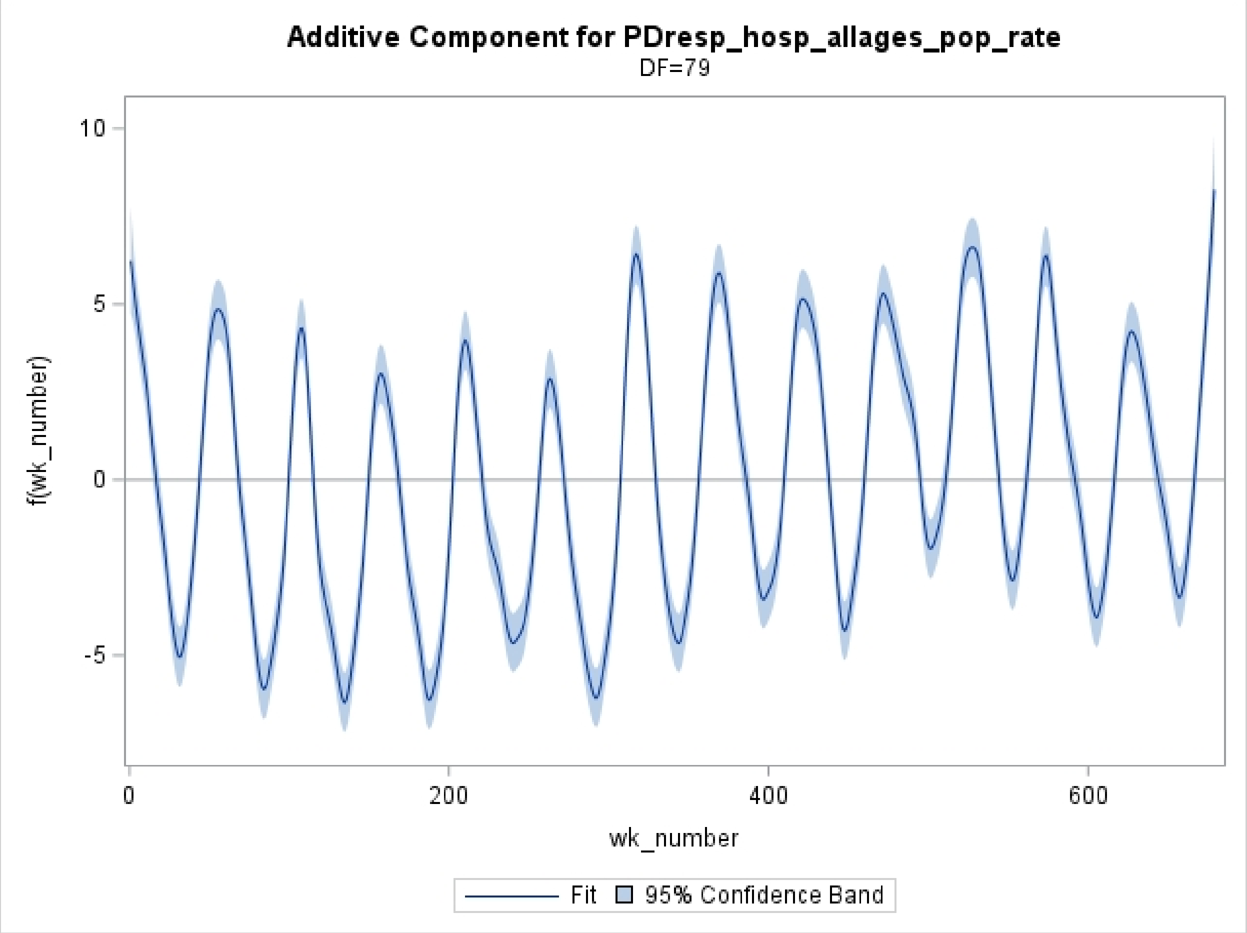

QQplot of residuals

The UNIVARIATE Procedure

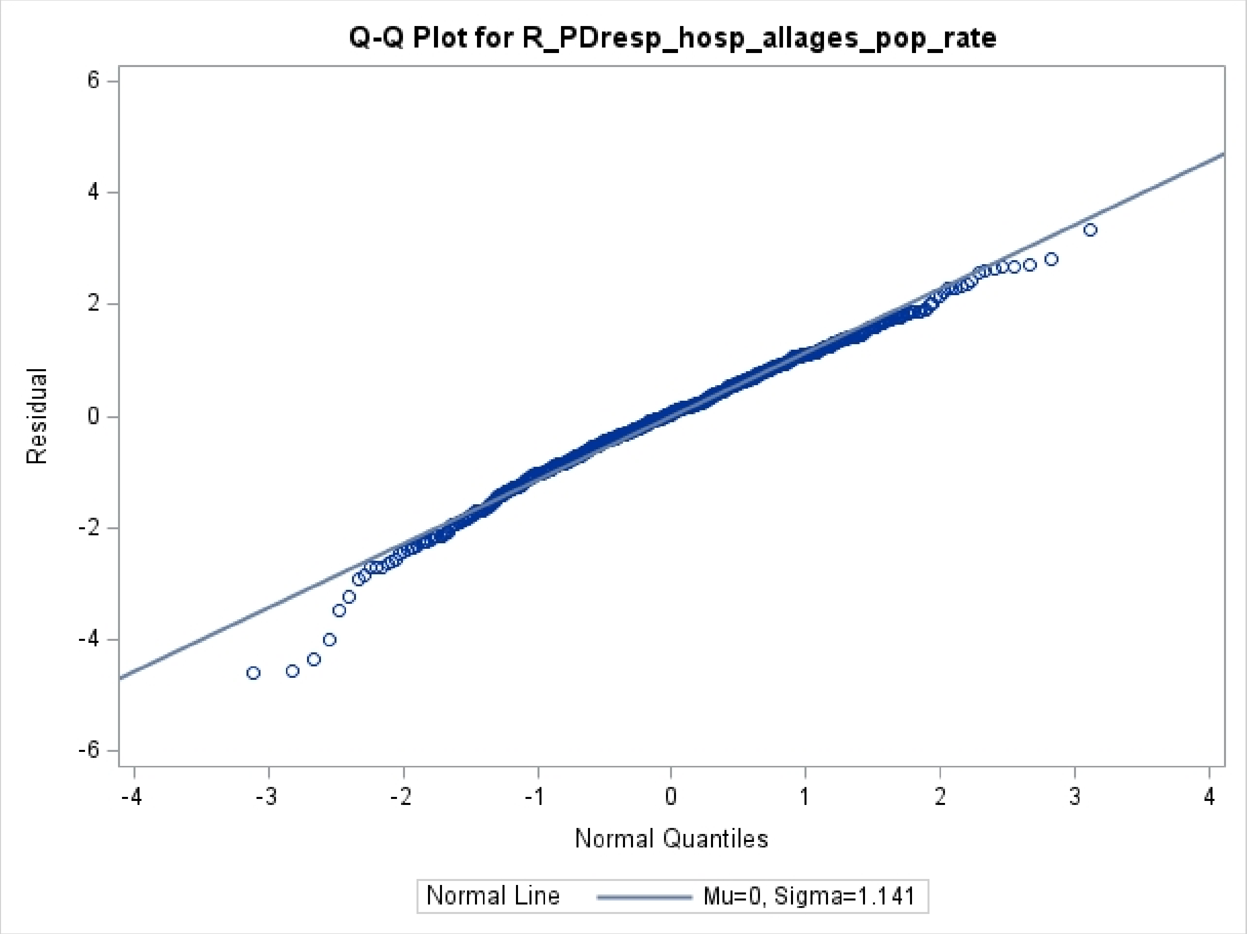

Autocorrelation plot of residuals

The ARIMA Procedure

Name of Variable = R_PDresp_hosp_allages_pop_rate	
Mean of Working Series	4.87E-16
Standard Deviation	1.140169
Number of Observations	679

Autocorrelation Check for White Noise									
To Lag	Chi-Square	DF	Pr > ChiSq	Autocorrelations					
6	76.99	6	<.0001	0.178	-0.080	-0.203	-0.158	-0.091	-0.002
12	125.41	12	<.0001	-0.131	-0.147	-0.088	-0.019	0.023	0.151
18	134.53	18	<.0001	0.072	0.061	0.003	-0.016	0.050	0.037
24	150.91	24	<.0001	-0.036	-0.093	-0.094	-0.053	0.003	0.042

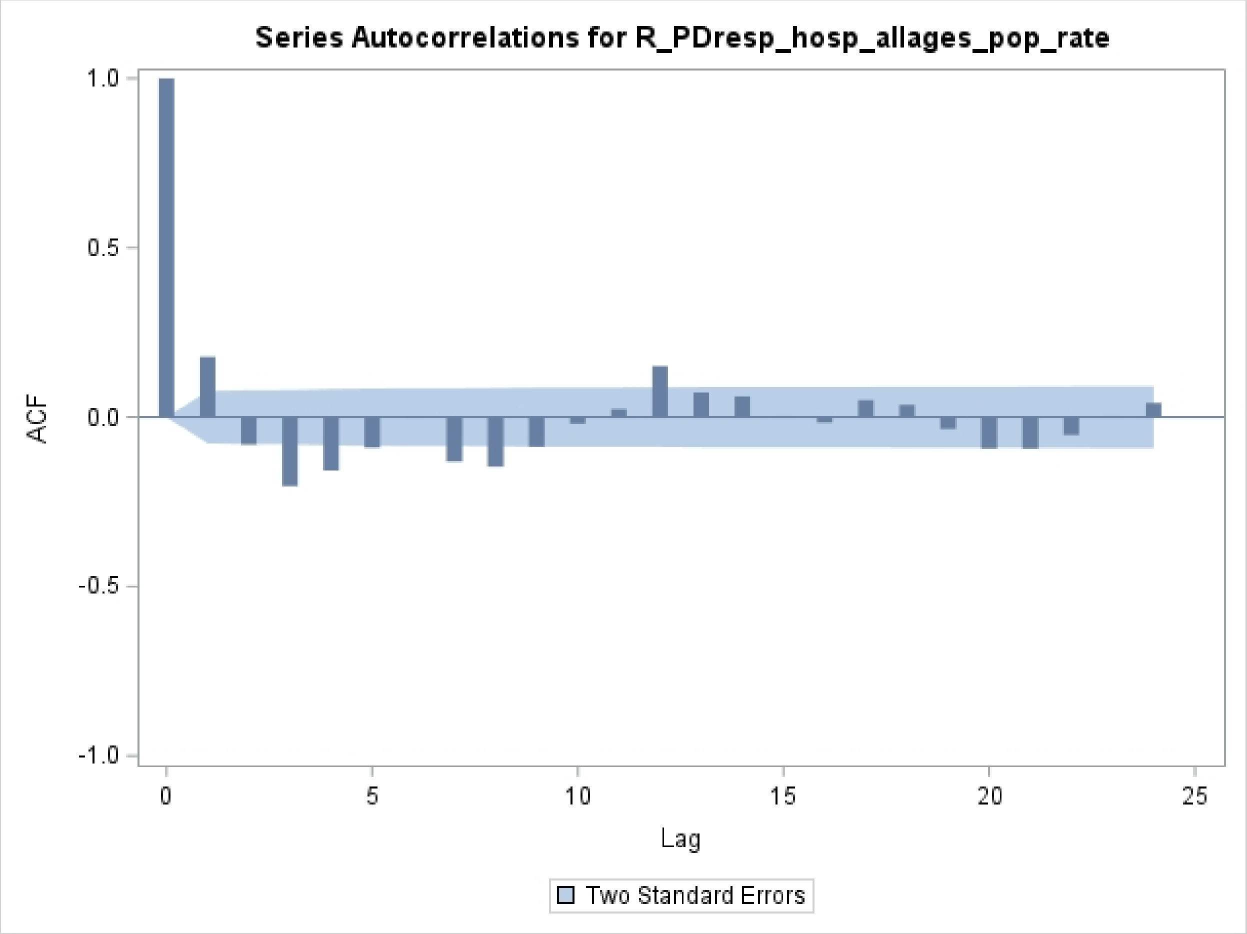

Supplement: S1 File — (PDF) [file pone.0230705.s009.pdf]
