# Supplementary material for: Estimated hospitalisations attributable to seasonal and pandemic influenza in Australia: 2001- 2013
Source: PLoS One. 2020 Apr 13;15(4):e0230705. doi: 10.1371/journal.pone.0230705 (PMC7153886; doi:10.1371/journal.pone.0230705)

GAM model output for all respiratory hospitalisations in persons of all ages, by total influenza

The GAM Procedure
Dependent Variable: PDresp_hosp_allages_pop_rate
Regression Model Component(s): week_before_Xmas Xmas_week new_year_week 1week_after_NY 2weeks_after_NY 3weeks_after_NY week_Australia_day one_week before_easter easter_week week_other_publicHday_Azday totalnoti_var_2001 totalnoti_var_2002 totalnoti_var_2003 totalnoti_var_2004 totalnoti_var_2005 totalnoti_var_2006 totalnoti_var_2007 totalnoti_var_2008 totalnoti_var_2009 totalnoti_var_2010 totalnoti_var_2011 totalnoti_var_2012 totalnoti_var_2013 totalnoti_var_2014
Smoothing Model Component(s): spline(wk_number)

Summary of Input Data Set	
Number of Observations	679
Number of Missing Observations	0
Distribution	Gaussian
Link Function	Identity

Iteration Summary and Fit Statistics	
Final Number of Backfitting Iterations	172
Final Backfitting Criterion	9.053673E-16
The Deviance of the Final Estimate	900.32725884

The backfitting algorithm converged.

Regression Model Analysis
Parameter Estimates

Parameter	Parameter Estimate	Standard Error	t Value	Pr > |t|
Intercept	30.44110	0.12895	236.07	<.0001
week_before_Xmas	-1.66548	0.34052	-4.89	<.0001
Xmas_week	-7.65344	0.35321	-21.67	<.0001
new_year_week	-6.98613	0.35317	-19.78	<.0001
1week_after_NY	-4.42713	0.35296	-12.54	<.0001
2weeks_after_NY	-3.24348	0.35291	-9.19	<.0001
3weeks_after_NY	-1.68049	0.39369	-4.27	<.0001
week_Australia_day	-3.75110	0.39374	-9.53	<.0001
one_week before_easter	-1.59154	0.35309	-4.51	<.0001
easter_week	-1.60643	0.36054	-4.46	<.0001
week_other_publicHday_Azday	-2.89731	0.36059	-8.03	<.0001
totalnoti_var_2001	0.04403	0.00433	10.17	<.0001
totalnoti_var_2002	0.02677	0.00163	16.46	<.0001
totalnoti_var_2003	0.03459	0.00126	27.35	<.0001
totalnoti_var_2004	0.04203	0.00291	14.46	<.0001
totalnoti_var_2005	0.01886	0.00140	13.45	<.0001
totalnoti_var_2006	0.02412	0.00184	13.08	<.0001
totalnoti_var_2007	0.00642	0.00043752	14.66	<.0001
totalnoti_var_2008	0.00932	0.00057982	16.07	<.0001
totalnoti_var_2009	0.00095107	0.00007588	12.53	<.0001
totalnoti_var_2010	0.00223	0.00043321	5.14	<.0001
totalnoti_var_2011	0.00124	0.00025081	4.96	<.0001
totalnoti_var_2012	0.00218	0.00012964	16.83	<.0001
totalnoti_var_2013	0.00300	0.00025199	11.91	<.0001
totalnoti_var_2014	0.00032976	0.00071723	0.46	0.6459
Linear(wk_number)	0.00425	0.00033477	12.70	<.0001

The GAM Procedure
Dependent Variable: PDresp_hosp_allages_pop_rate
Regression Model Component(s): week_before_Xmas Xmas_week new_year_week 1week_after_NY 2weeks_after_NY 3weeks_after_NY week_Australia_day one_week before_easter easter_week week_other_publicHday_Azday totalnoti_var_2001 totalnoti_var_2002 totalnoti_var_2003 totalnoti_var_2004 totalnoti_var_2005 totalnoti_var_2006 totalnoti_var_2007 totalnoti_var_2008 totalnoti_var_2009 totalnoti_var_2010 totalnoti_var_2011 totalnoti_var_2012 totalnoti_var_2013 totalnoti_var_2014
Smoothing Model Component(s): spline(wk_number)

Smoothing Model Analysis
Fit Summary for Smoothing Components

Component	Smoothing Parameter	DF	GCV	Num Unique Obs
Spline(wk_number)	0.994167	78.000000	1.703792	679

Smoothing Model Analysis
Analysis of Deviance

Source	DF	Sum of Squares	Chi-Square	Pr > ChiSq
Spline(wk_number)	78.00000	4487.751847	2866.1326	<.0001

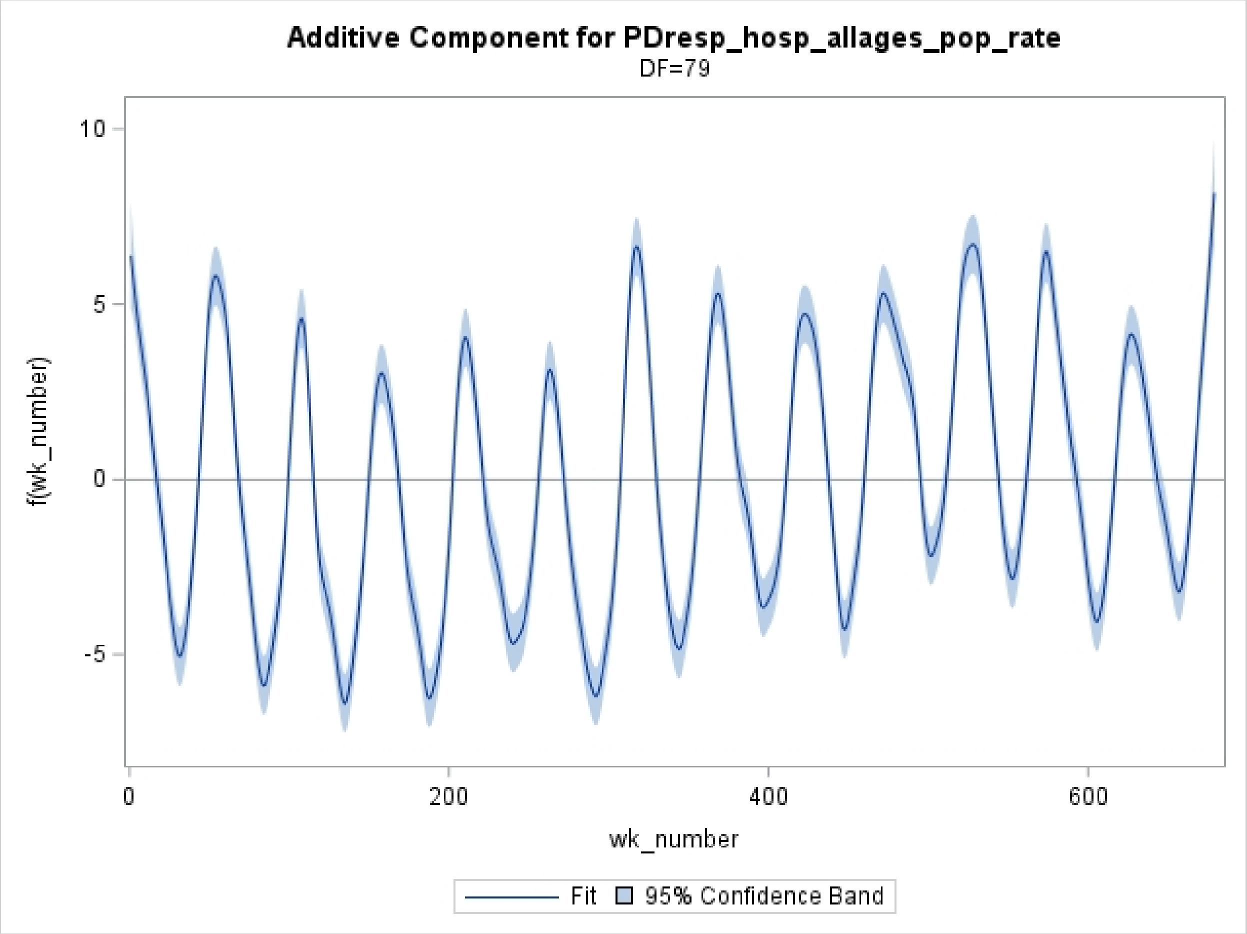

QQplot of residuals

The UNIVARIATE Procedure

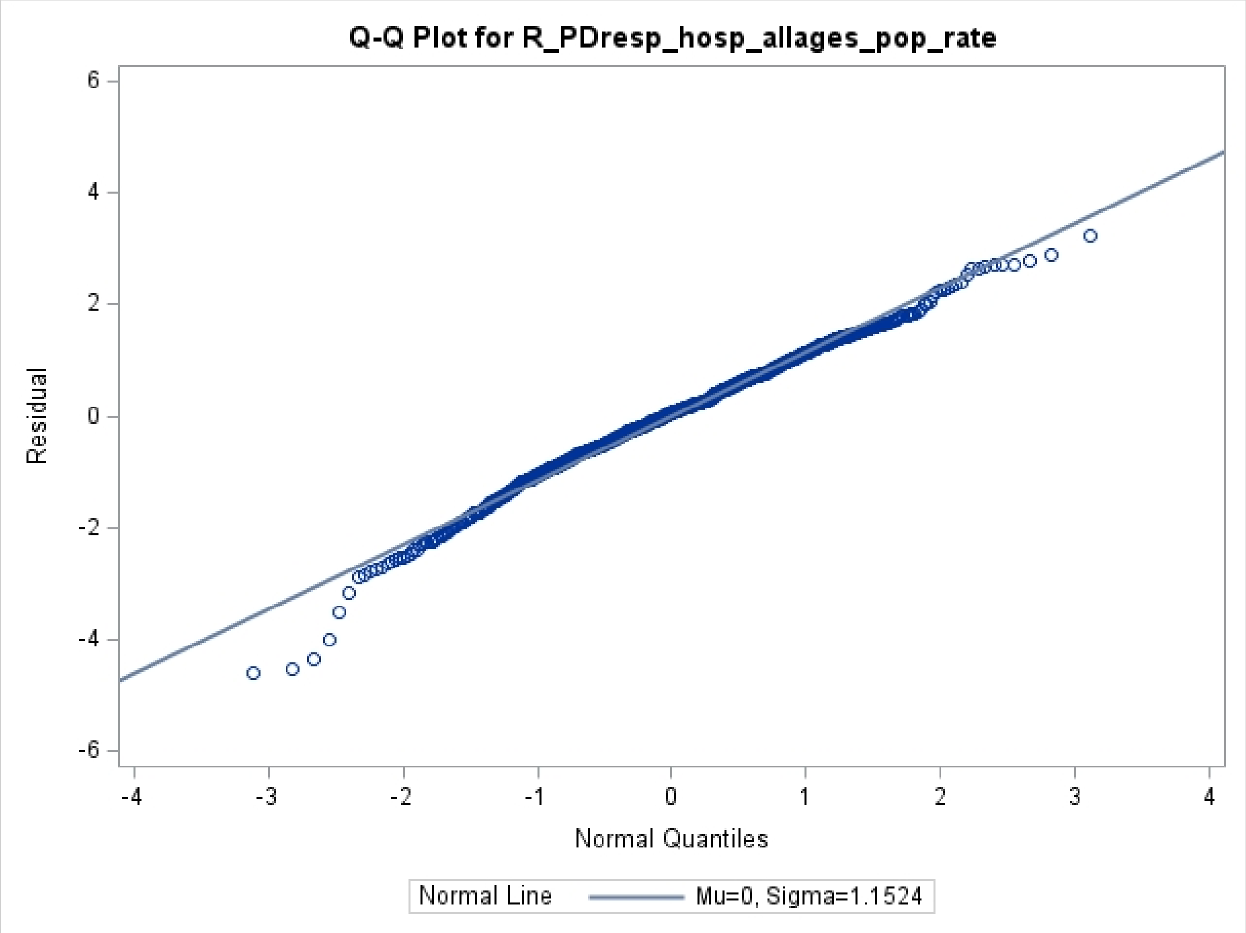

Autocorrelation plot of residuals

The ARIMA Procedure

Name of Variable = R_PDresp_hosp_allages_pop_rate	
Mean of Working Series	-28E-16
Standard Deviation	1.151504
Number of Observations	679

Autocorrelation Check for White Noise									
To Lag	Chi-Square	DF	Pr > ChiSq	Autocorrelations					
6	81.96	6	<.0001	0.194	-0.068	-0.205	-0.155	-0.106	-0.014
12	135.72	12	<.0001	-0.135	-0.152	-0.107	-0.022	0.038	0.153
18	146.02	18	<.0001	0.086	0.059	0.008	-0.009	0.053	0.032
24	165.00	24	<.0001	-0.036	-0.105	-0.096	-0.061	-0.008	0.043

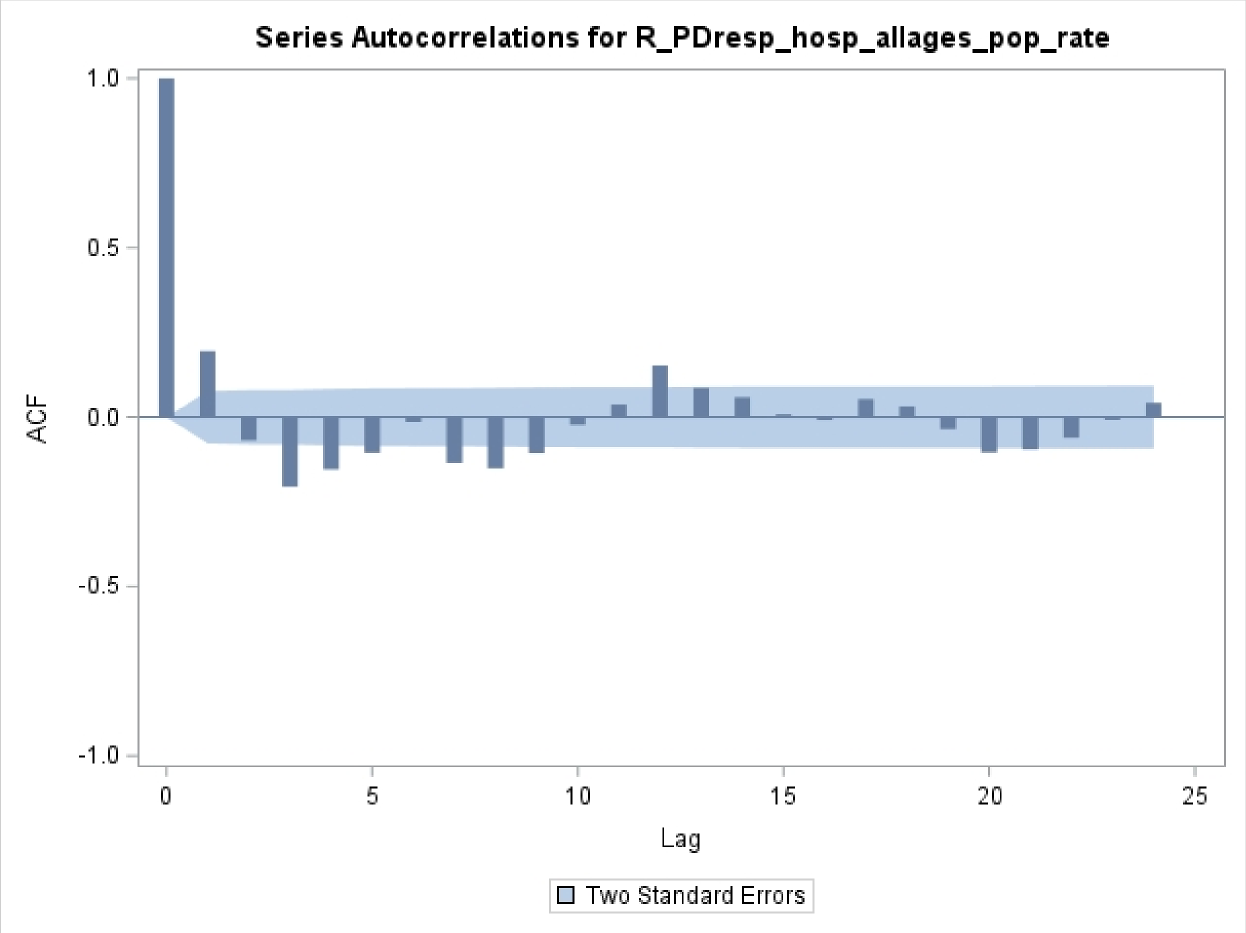

Supplement: S2 File — (PDF) [file pone.0230705.s010.pdf]
